# Duropathies as Unifying Concept—Part Two: A Narrative Overview of Clinical and Neuroradiological Features

**DOI:** 10.3390/neurolint18030060

**Published:** 2026-03-20

**Authors:** Marialuisa Zedde, Luigi Cirillo, Elisa Francesca Maria Ciceri, Nicola Limbucci, Mario Muto, Mauro Bergui, Francesco Causin, Rosario Pascarella

**Affiliations:** 1 Neurology Unit, Stroke Unit, Azienda Unità Sanitaria Locale-IRCCS di Reggio Emilia, Viale Risorgimento 80, 42123 Reggio Emilia, Italy; 2 Neuroradiology Unit, IRCCS Institute of Neurological Sciences of Bologna, 40124 Bologna, Italy; 3 Department of Biomedical and Neuromotor Sciences (DIBINEM), University of Bologna, 40126 Bologna, Italy; 4 Neurointerventional Radiology Unit, Fondazione IRCCS Istituto Neurologico Carlo Besta, 20133 Milan, Italy; 5 Interventional Neurovascular Unit, Careggi University Hospital, 50134 Florence, Italy; 6 Diagnostic and Interventional Neuroradiology Unit, Cardarelli Hospital, 80131 Naples, Italy; mutomar2@gmail.com; 7 Department of Neuroscience, Neuroradiological Unit, University of Turin, Azienda Ospedaliera Città Della Salute E Della Scienza Hospital, 10126 Turin, Italy; 8 AOPD UOC Neuroradiologia, Azienda Ospedale Università Di Padova, 35128 Padua, Italy; francesco.causin@aopd.veneto.it; 9 Neuroradiology Unit, Ospedale Santa Maria della Misericordia, AULSS 5 Polesana, 45100 Rovigo, Italy

**Keywords:** superficial siderosis, spontaneous intracranial hypotension, dural tear, CSF leak, ventral spinal fluid collection, spinal cord herniation, spinal arachnoid web, CT myelography, MRI

## Abstract

Duropathies represent a spectrum of disorders associated with spinal dural tears and cerebrospinal fluid (CSF) leaks. Diagnosis and treatment is often complicated by overlapping clinical manifestations. This review aims to synthesize current literature on duropathies, focusing on their clinical, neuroradiological, and pathophysiological features. A comprehensive literature review was conducted, analyzing various conditions classified as duropathies, including spontaneous intracranial hypotension (SIH), superficial siderosis (SS), spinal cord herniation, and, as added issue, arachnoid webs. The review emphasized the importance of imaging techniques such as MRI and CT myelography in diagnosing these conditions. Duropathies can arise from congenital anomalies, trauma, and degenerative changes, with SIH being characterized by orthostatic headaches and neurological deficits. Imaging typically reveals specific patterns, such as a widened dorsal subarachnoid space and ventral displacement of the spinal cord. Syringomyelia was frequently associated with arachnoid webs, and complications like SS and bibrachial amyotrophy were noted in patients with persistent ventral spinal CSF leaks. The unifying concept of duropathies is proposed, emphasizing the need for timely intervention to mitigate long-term neurological consequences. Enhanced diagnostic strategies are crucial for improving patient outcomes, and a multidisciplinary approach is recommended for the management of these complex disorders. Further research is warranted to clarify the pathophysiological mechanisms underlying duropathies and to establish standardized treatment protocols.

## 1. Introduction

The term “duropathies” was first introduced by Kumar et al. [1] to describe a group of disorders linked by a common pathogenetic trigger: a spinal dural tear accompanied by a cerebrospinal fluid (CSF) leak within the spinal dura mater. This category encompasses a wide clinical spectrum of conditions, including craniospinal hypovolemia (also known as spontaneous intracranial hypotension or SIH), which is frequently characterized by orthostatic headache, and superficial siderosis (SS), causing ataxia and auditory impairment. Additionally, patients may experience segmental weakness and atrophy, which can occur either with or without hyperreflexia, alongside the serious complication of spinal cord herniation [2]. Notably, the clinical manifestations of these disorders often overlap, complicating diagnosis and treatment. A significant feature present across all these conditions is the dural defect–related ventral longitudinal intraspinal fluid collection (VLISFC), which plays a crucial role in the disease process [3].

Duropathies can originate from various causes, such as injuries, congenital defects, and degenerative processes affecting the spine. The main pathophysiological hallmark of SIH is the decrease in CSF volume, often due to tears in the dura mater, leading to severe orthostatic headache and other neurological symptoms, underscoring the essential function of the meninges in regulating CSF balance and overall neurological health. Subarachnoid hemorrhage, which results from persistent bleeding in the subarachnoid space, can cause the accumulation of hemosiderin, leading to progressive neurological deterioration, particularly affecting the cerebellum and auditory pathways. Another notable consequence of dural disorders is spinal cord herniation, which occurs when the spinal cord extends through a defect in the dura, frequently resulting in significant symptoms like muscle weakness and sensory issues. Furthermore, arachnoid webs, recently proposed as a component of duropathies, can apply pressure on the spinal cord, complicating the clinical scenario by hindering normal CSF circulation and contributing to conditions such as syringomyelia.

A thorough understanding of the fundamental mechanisms and clinical consequences of duropathies is crucial for reaching the effective diagnostic and deriving standardized treatment approaches. A recent review examined the anatomical, embryological, and pathophysiological issues of duropathies, being the ideal premise to the clinical and neuroradiological approach to the diagnosis that is the topic of the present paper [4]. In fact, this review aims to consolidate existing research on these conditions, offering a detailed examination of the clinical and neuroradiological findings supported by the involved pathophysiological factors. A thorough understanding of the clinical and imaging characteristics related to duropathies can promote a cohesive multidisciplinary framework for further research and standardized management. A detailed description of surgical treatment is outside the aim of this review.

## 2. Methods

We propose a narrative review with illustrative examples of the main neuroimaging issues in patients with duropathies. This topic has not been systematically addressed in the literature, and the heterogeneity of the available data prevents addressing it with a systematic review. We thus performed a literature search using PubMed starting from January 2000 and using the general term “duropath*” and retrieved 14 papers. We checked the references proposed by all of them and performed a second step of literature search for each condition:

- (spontaneous intracranial hypotension) AND (dural leak): 357 records
- (superficial siderosis) AND (dural leak): 32 records
- (spinal cord herniation) AND (dural leak): 24 records
- (arachnoid web) AND (dural leak): 3 records
- (bibrachial amyotrophy) AND (dural leak): 2 records.

We sought for back and forward citations of each paper and we selected the most informative papers describing clinical, neuroimaging, and pathological data for the current narrative review.

The primary aim of this narrative review is to consolidate and describe in an updated and comprehensive text the available information on this topic, focusing on both clinical and neuroradiological issues and using pathological information to derive some pathophysiological hypotheses. This paper is the second step of a previous review focused on anatomic and embryologic issues [4].

## 3. The Spectrum of Duropathies: Clinical and Pathophysiological Features

**Figure 1:**
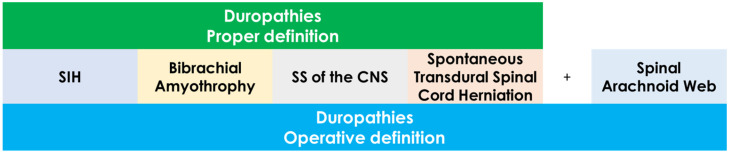
The proposed evolving concept of duropathies encompasses the diseases included in the proper definition to include other diseases (i.e., spinal arachnoid web) in an operative definition.

Dural abnormalities are commonly found in cases of infratentorial SS, with over 80% of patients showing dural tears as the underlying cause, especially in individuals with spinal siderosis and VLISFC [5]. This correlation has given rise to the term “duropathies,” which includes conditions like SS of the central nervous system (CNS), SIH, multisegmental amyotrophy, and spinal cord herniation. Nevertheless, the pathophysiology and connections between these overlapping duropathies remain ambiguous. Although the specific type of dural defect can help clarify the symptoms, it is still unclear why some patients display only a single manifestation of the duropathy spectrum, while others may show multiple conditions. In addition, some conditions, such as spinal arachnoid web, may share pathophysiological, clinical and neuroradiological features with duropathies and they can be pragmatically included into duropathies (Figure 1).

In the next paragraphs, the diseases included in the operative definition of duropathies are individually addressed from the clinical and pathophysiological point of view, aiming to highlight the common shared features and the overlapping phenotype in the natural history of these diseases.

### 3.1. Spontaneous Intracranial Hypotension

As previously introduced, SIH is a condition marked by a reduced CSF volume due to CSF leakage [6]. While it is generally characterized by a low CSF opening pressure (<6 cm of water), a diagnosis is not ruled out if the opening pressure is normal, particularly after more than 3 weeks from the symptoms onset; further investigation should continue if convincing clinical symptoms and imaging abnormalities are present [7]. SIH can be differentiated from other causes of intracranial hypotension, including craniospinal trauma, spinal surgery, lumbar puncture, and overshunting. The incidence of SIH is estimated at 5 per 100,000 individuals, peaking around the age of 40, and it tends to affect women more frequently [8]. The three primary sources of CSF leaks are: (1) tears in the spinal dural membrane, (2) rupture of a meningeal diverticulum, and (3) CSF-venous fistulas [9,10]. The classification of SIH is based on the source of the leak [9]. Most spontaneous CSF leaks are spinal, particularly at the thoracic level or cervicothoracic junction, with less frequent occurrences at the skull base and sacral regions [11,12,13].

Postural headache is the hallmark symptom of SIH, as outlined in the International Classification of Headache Disorders (ICHD, 3rd Edition), which defines it as a headache linked to low CSF pressure or CSF leakage [14]. Nonetheless, the clinical presentations of SIH can vary widely. A systematic review and meta-analysis of 144 studies revealed that a small proportion of patients might experience headaches unrelated to posture (8%), normal findings on lumbar punctures (normal opening pressure and benign CSF analysis) (32%), or unremarkable imaging results (19%) [15]. Common accompanying symptoms include nausea and vomiting, neck pain and stiffness, tinnitus, dizziness, and cognitive dullness. The diagnostic criteria require objective evidence of SIH, which may involve brain Magnetic Resonance Imaging (MRI) findings suggestive of the condition (such as pachymeningeal enhancement, brain sagging, or subdural fluid collections), spinal imaging that shows a CSF leak (like the presence of extradural CSF collection or a CSF-venous fistula), or a low CSF opening pressure (i.e., less than 6.0 cm H_2_O) [14,15]. These criteria are generally applied even to patients without headache when their symptoms can be attributed to SIH, although not validated. A diagnosis of a ventral spinal CSF leak can be established through radiographic techniques if the extrathecal CSF collection is localized to the ventral side of the common thecal sac or if a ventral dural tear is identified via digital subtraction myelography (DSM) or dynamic CT myelography, even when the extrathecal CSF collection is circumferential.

Given the variability in clinical presentations [15], employing a systematic approach for diagnosing and managing these patients is essential for facilitating quicker diagnosis and treatment. It is vital to recognize the imaging findings associated with SIH and spinal CSF leaks, as well as the indications and methods for identifying, localizing, and treating CSF leaks. Spinal MRI plays a crucial role in the diagnostic pathway of SIH. The diagnostic myelographic evaluation is contingent upon the presence or absence of a spinal longitudinal extradural collection (in particular VLISFC), which determines whether to proceed with dynamic CT myelography or lateral decubitus DSM. Notably, up to 33% of SIH cases may not show epidural CSF collections on multimodal imaging [16]. Dural tears are the most common cause of SIH, but they are variably associated with the previously mentioned conditions. In fact, SS, bibrachial amyotrophy, and spinal cord herniation are rare yet serious long-term consequences of ongoing spontaneous spinal CSF leaks in SIH patients, particularly those with VLISFCs [17]. In fact, SS [18,19,20,21], bibrachial amyotrophy [22,23,24,25], and spinal cord herniation [26,27] predominantly occur in patients with longstanding ventral (i.e., type 1a) spinal CSF leaks [18,19,20,21,22,23,24,25,26,27]. Approximately 25% of patients with SIH present with a ventral spinal CSF leak [9]. Due to their ventral position relative to the spinal cord and the nearly constant presence of an associated bony spur [9,28,29]. They have a trend to become chronic and potentially persist for years or decades, despite a gradual reduction in headache severity and the ability for patients to return to a normal or nearly normal lifestyle. A cohort study sought to assess the risk of these severe complications stemming from persistent VLISFCs in a high-volume center with a prospective registry of SIH patients starting in 2001 [17]. In this study, individuals with VLISFCs who were first evaluated within one year of symptom onset were included, excluding those who had successful leak repairs, resulting in a final sample of 51 patients [17]. The authors reported a chance of developing SS and/or bibrachial amyotrophy during the follow-up increasing with the duration of the follow-up: 0% at 8 months, 4.5% (95% CI 1.0–28.0%) at 56 months, 10.5% (95% CI 3.0–36.4%) at 96 months, 32.7% (95% CI 15.0–62.8%) at 144 months, and 57.9% (95% CI 30.2–87.6%) at 192 months. SS was asymptomatic in 4/6 (66%) patients and symptomatic for isolated hearing loss and hearing loss with ataxia in the remaining two patients, showing a more extensive SS than the asymptomatic ones. According with these observational data, the authors postulated that an early intervention for resolving the dural tear and the VLISFC could prevent a chronic neurological impairment due to the development of SS and bibrachial amyotrophy [17], but the lack of prospective randomized trials to support this conclusion does not allow to recommend this approach, although treatment for VLISFCs is associated with minimal morbidity [30,31]. Furthermore, patients with a history of SIH, especially those with extradural CSF collections, should be considered for brain and spinal MRI in the follow-up, even if they exhibit only mild or no symptoms.

A more recent study from the same group aimed to assess the risk of spinal cord herniation in SIH patients with VLISFC, reporting a relatively low rate (3.9% of patients after a median follow-up of 86.1 months) [32].

### 3.2. Bibrachial Amyotrophy

Bibrachial amyotrophy, which is linked to SIH, is marked by a gradual and painless weakening and atrophy of the shoulder and upper limb muscles. This condition often presents with fasciculation and amyotrophic lateral sclerosis (ALS) is among the differential diagnoses [23,24,25]. In individuals with ongoing spinal CSF leaks, bibrachial amyotrophy is thought to result from the stretching of cervical nerve roots due to the presence of an extradural CSF collection. This pathophysiological hypothesis is supported by the finding of significant enlargement of the extradural CSF collection over time in patients who went on to develop bibrachial amyotrophy [17].

However, bibrachial amyotrophy, associated with VLISFCs and infratentorial SS, is a rare condition and both diseases can sometimes clinically resemble motor neuron diseases [5,33]. Classic infratentorial SS is characterized by the deposition of hemosiderin in the subpial layers of the brain and spinal cord, which can lead to sensorineural hearing loss, cerebellar ataxia, and myelopathy. Approximately 5–10% of cases exhibit features of lower motor neuron (LMN) involvement, including muscle wasting [33]. Since its initial identification in 1908 [34], only a limited number of cases have been documented that align with a motor neuron disease phenotype [35,36,37,38,39,40,41]. Infratentorial SS mainly arises from a dural defect that causes ongoing low-volume bleeding into the subarachnoid space due to fragile blood vessels [5,18,42]. Additionally, VLISFC is frequently found across multiple spinal levels [18,20]. SIH includes three recognized types of CSF leaks [9], with SS predominantly found in patients with type 1a dural defects—characterized by a small ventral dural slit due to degenerative spinal conditions—and occasionally in those with type 2 defects (meningeal diverticula) [8,43]. The first report connecting dural leaks, VLISFC, and damage to anterior horn cells was published in 2011 [22]. A recent review on bibrachial amyotrophy as a rare consequence of VLISFC described 44 patients, of whom only 39% were associated with SS [44]. In another study, SS was present in 9% of patients with CSF leaks [42]. As previously detailed, in individuals with chronic dural leaks and SIH, the risk of developing SS was around 50%, while the likelihood of bibrachial amyotrophy was about 20% after 15 years of follow-up [17]. Although a well-established connection exists between SIH and SS—typically with SIH occurring before SS—some patients with SS lack a history of symptoms related to SIH. Historically, LMN involvement in infratentorial SS was thought to be due to arachnoiditis or radiculopathy, suggesting that hemosiderin deposition occurs at the motor nerve root exit zone [38] or directly at the level of the anterior horn cells. Conversely, for bibrachial amyotrophy associated with VLISFC, two potential mechanisms have been proposed: (1) compression of anterior horn cells due to VLISFC and/or (2) stretching of the motor nerve roots from posterior displacement of the spinal cord by the fluid collection [23,25,43]. Pathological analysis of a case of bibrachial amyotrophy associated with SS and a dural tear suggested that VLISFC plays a crucial role in anterior horn damage, ruling out hemosiderin deposition as the primary cause [44]. Notably, nearly all reported cases of infratentorial SS presenting with an ALS-like phenotype had VLISFC, supporting this theory [36,41]. Furthermore, there are no known instances of infratentorial SS occurring prior to the onset of bibrachial amyotrophy. On the other side, symptoms of SIH may manifest after the onset of bibrachial amyotrophy in VLISFC cases [45]. In these instances, the time between the beginning of weakness and the development of orthostatic headaches was as long as 26 months.

However, brachial amyotrophy is an exceptionally rare phenotype found in patients with SS and VLISFC [21]. The depth of SS penetration in brain and spinal cord tissue has been pathologically defined as 3 mm, making it difficult to justify anterior horn damage as a cause of a motor neuron pattern in these patients, diverging from the mechanisms described for VLISFC and bibrachial amyotrophy [46,47,48]. The “snake-eyes” appearance observed in cervical spinal cord MRI has been reported in patients with bibrachial amyotrophy linked to cervical spondylosis or ossification of the posterior longitudinal ligament (OPLL) [49,50]. Pathological examination of an autopsied patient with OPLL and the snake-eyes appearance on MRI revealed intramedullary cystic necrosis surrounding the central gray matter and the ventrolateral posterior column, along with a loss of anterior horn cells [49]. Autopsy case series involving cervical spondylotic myelopathy have shown similar neuropathological findings, indicating that atrophy and neuronal loss generally initiate in the anterior horns and intermediate zone of the spinal gray matter [48]. The pathophysiology of compressive spinal cord injury has been hypothesized to stem from circulatory disturbances [50,51,52]. In a case described by Takahashi et al. [47], the snake-eyes appearance on MRI and the pattern of spinal cord damage observed in pathological analysis of one patient with VLISFC and bibrachial amyotrophy were akin to findings seen in patients with compressive myelopathy [50,51]. The histological similarities between the spinal gray matter in spondylotic myelopathy and Takahashi’s case [47,50] suggest that VLISFC plays a crucial role in the development of anterior horn damage. However, in the above-cited case the volume of VLISFC was too small to cause significant compression of the cervical spinal cord on MRI [47]. In contrast, the volume of VLISFC was greater at the thoracic spinal cord level, particularly in the middle thoracic region [47]. The reasons for the discrepancy between the significant loss of anterior horn cells in the middle cervical to upper thoracic cord and the largest volume of VLISFC at the middle thoracic cord remain unclear. It is possible that the greater range of motion in the cervical spine compared to the thoracic spine may contribute to this discrepancy. In Hirayama disease, an MRI taken in a neck flexion position can show compression of the cervical spinal cord due to forward displacement of the cervical dural sac [53]. Regarding the theory that VLISFC may stretch the motor nerve roots, the extensive damage observed in the anterior horns and intermediate zone, characterized by the snake-eyes appearance on MRI [50], is unlikely to be solely attributed to motor nerve root damage. In addition, the widespread superficial hemosiderin deposition throughout the spinal cord [50,51] does not support the notion that bibrachial amyotrophy is primarily caused by hemosiderin accumulation. However, some motor neurons exhibiting chromatolysis were noted in segments with preserved anterior horn cells; this finding may indicate axonal damage to these motor neurons due to hemosiderin deposition [50,51]. The absence of notable chromatolysis in segments with severe anterior horn damage may be due to the depletion of nearly all large neurons corresponding to motor neurons in those areas [47,50,51]. However, in some cases neurophysiological study shows both chronic and subacute neurogenic changes, reflecting damage not only in the anterior horns but also at the radicular level [42,44]. This damage is attributed to the stretching of motor roots caused by the posterior displacement of the spinal cord due to fluid collection. This mechanism has been proposed as a potential contributor to amyotrophy, resulting in secondary degeneration of anterior horn neural cells due to the displacement of motor roots. Oxidative stress associated with hemosiderin deposition may further exacerbate damage at the spinal level [37,42]. Interestingly, some patients with both bibrachial amyotrophy and VLISFC do not show SS on brain MRI [25]. For instance, Morishima et al. [25] reported a case without SS on brain MRI or the presence of red blood cells in the CSF. These findings suggest that hemosiderin deposition is not mandatory for the development of bibrachial amyotrophy in patients with VLISFC [54].

Nevertheless, some reports consider CSF leaks as a differential diagnosis for motor neuron diseases, highlighting the main features of the 44 patients documented in the literature [44]. The majority of these patients were male (38 out of 44), with a median age of 52.5 years (ranging from 22 to 79). A minority of cases (14 out of 44) reported a history of prior trauma or cranial/spinal surgery, with injuries typically occurring several years before the onset of neurological symptoms. The median duration of the disease, from onset to clinical observation, was 78 months (range 3–480). All patients reported progressive weakness as the initial symptom, although only 3 out of 44 (6.8%) mentioned a history of pre-existing orthostatic headaches. Most cases (33 out of 44) exhibited bilateral involvement of the upper limbs. Additionally, 27 patients presented with clinical features beyond amyotrophy, including ataxia (31.8%), hearing issues (25%), headache (18.2%), sensory disturbances (15.9%), and, in one atypical case, a clinical presentation resembling pseudotumor cerebri. Spinal MRI demonstrated high-signal-intensity lesions in the bilateral anterior horns at cervical levels (mostly C2–C6) for the majority of patients. In one case, atrophy with myelomalacia was reported only at the thoracic level. The VLISFC affected cervical-thoracic spinal tracts and, in some instances, extended to the lumbar spinal levels. A dural defect was identified in 23 patients (52.3%), primarily located at the thoracic spinal level. MRI findings indicated SS in 15 patients (34.1%), with two cases in the cervical spinal cord [44]. CSF opening pressures were assessed in 13 patients; most showed normal levels, with two exceptions: one patient had a pressure exceeding 50 cm H_2_O, while another had a pressure of 0 [44]. Interestingly, orthostatic headache is time-dependent in patients with bibrachial amyotrophy, as in patients with SIH. In a cohort study of patients with SIH and CSF leaks, those with shorter disease durations frequently reported headache and abnormally low CSF opening pressures. Conversely, the incidence of headache decreased in subacute and chronic cases, alongside the normalization of CSF pressure and dynamics, likely due to compensatory mechanisms that are not yet fully understood [53]. Additionally, one patient exhibited increased CSF pressure associated with symptoms of intracranial hypertension (diplopia, papilledema, bilateral VI nerve palsy, right partial III nerve palsy) [53]. However, the exact pathogenic mechanism for this co-occurrence remains unclear, and the connection to intracranial hypertension appears limited to this single case [39].

Another noteworthy finding is the association with SS in approximately 39% of the reported patients, who often exhibited additional features (14/17), primarily ataxia and hearing loss, aligning with the spectrum of SS of the CNS [37,42]. This is not unexpected, considering that SS has been documented in 9% of cases with CSF leaks, with 43% of those also presenting with bibrachial amyotrophy [42].

Although some cases of bibrachial amyotrophy have been misdiagnosed as ALS or other motor neuron diseases [37,39,55,56], this condition generally presents with an earlier onset and a slower progression compared to ALS, often without evident upper motor neuron involvement. In this context, progressive muscular atrophy (PMA) might be included in the differential diagnosis, although PMA is typically diagnosed at an older age than ALS and tends to progress more quickly than localized amyotrophy [57]. Furthermore, among benign forms of amyotrophy, Hirayama disease [52,57] poses diagnostic challenges due to its similarities with this condition. Hirayama syndrome usually presents with unilateral or bilateral weakness in the forearms and hands, typically without pyramidal signs. It also exhibits high-signal hyperintensities in the cervical spinal cord, which are attributed to chronic ischemic microchanges affecting anterior horn cells due to reduced thecal sac laxity during cervical flexion [57]. However, Hirayama syndrome primarily affects juveniles and typically progresses gradually over a period of 1 to 4 years before stabilizing.

### 3.3. Spontaneous Transdural Spinal Cord Herniation

Spontaneous transdural spinal cord herniation (STSCH) is a rare condition defined by the protrusion of the spinal cord through a defect in the dura mater [26,27,58,59]. Typically, this defect is found anteriorly or anterolaterally. Patients with STSCH commonly exhibit neurological deficits ranging from spastic paraparesis with progressive neurological deterioration to Brown–Sequard syndrome. While the etiology of STSCH remains debated, the predominant theory posits that it arises from an initial dural tear followed by gradual herniation of the spinal cord through this tear. A follow-up study of SIH patients with ventral dural tears [17] found no cases of spinal cord herniation during the follow-up period, which should not be interpreted as evidence of a congenital cause. Instead, this likely reflects the rarity of such herniation events. Alternatively, early tamponade of the dural defect by the spinal cord might result in milder symptoms of SIH, leading patients to seek medical attention less frequently, thereby potentially increasing the risk for herniation. In fact, a further study reported a 3.9% rate of STSCH in patients with SIH and VLISFC after a median follow-up of 86.1 months [32].

First described by Wortzman et al. in 1974 [60,61,62], STSCH is a rare but potentially treatable cause of thoracic myelopathy, predominantly affecting middle-aged adults with a female predominance and a predilection for the thoracic involvement [60,61,62,63]. Several theories have been proposed to explain the pathogenesis of STSCH; however, the exact origin of the dural defects remains unclear [60]. Surgical intervention aimed at repositioning the herniated cord back into its normal intradural location is crucial for maximizing neurological recovery. The primary surgical techniques employed to address STSCH include dural defect enlargement and duraplasty. In recent years, the increasing incidence of STSCH has led to a rise in surgical procedures. A well-defined set of MRI diagnostic criteria for STSCH has been established [64,65,66], although guidelines for interpreting postoperative MRI changes in the spinal cord and surrounding structures are lacking. A limited understanding of postoperative MRI results and their temporal evolution can lead to misinterpretations of normal postoperative changes and the oversight of potential complications [64,65,66].

The true incidence of STSCH remains uncertain. A study involving 15,805 patients found that only 12 (0.08%) underwent surgery for SISCH, confirming its rarity [65]. Consistent with previous findings, STSCH primarily affects middle-aged and elderly individuals, with a slightly higher prevalence in females. The upper-middle thoracic region is identified as the most commonly affected area. Potential origins of STSCH may include congenital anomalies, minor trauma, and arachnoid cysts [65]. However, a definitive pathology has yet to be established. Notably, 67% of patients were classified as having a spinal cord herniation at the disc level, although complications related to disc displacement were not observed [65]. Intraoperative findings revealed a duplicated dura in 92% of patients, and pathological examinations showed no evidence of dural inflammation [65]. The gradual onset of myelopathy, typically limited to the thoracic spine, may be attributed to a weakened or thinner ventral dura. This could result from congenital or acquired factors that lead to duplicated dura and cerebrospinal fluid pulsation, ultimately causing a longitudinal defect that result in STSCH [65]. Regarding bone defects, no relationship was found with disease duration or age; however, patients with bone defects presented with more severe preoperative symptoms [65]. The lack of correlation with disease duration and age suggests that those with bone defects may experience easier herniation. The repeated pulling of the spinal cord due to pulsation may exacerbate spinal disorders and symptoms more rapidly compared to patients with a dural defect alone.

However, spontaneous ventral herniation of the spinal cord through a dural defect, even in the absence of prior injury, is an extremely rare phenomenon with poorly understood pathophysiology. In 1991, Isu et al. [67] proposed that ventral defects could arise from pressure exerted by the cord due to a dorsal arachnoid cyst, noting some sensory improvement after cyst excision. However, this explanation seems improbable in subsequent cases. Arachnoid cysts have been associated with spinal cord herniation in 14 out of 79 cases, primarily in earlier reports [58]. Thickened arachnoid tissue, which raised the possibility of an arachnoid cyst, has been noted in several instances [58], but it likely does not significantly contribute to the pathophysiology of the disease, with misdiagnoses stemming from misinterpretation of thickened arachnoid tissue [68]. While a few cases have noted a remote history of trauma [58], linking dural defects and herniation to such distant events is challenging, given the low likelihood of significant trauma causing a dural tear without neurologic injury. Most traumatic and iatrogenic spinal cord herniations tend to be posterior rather than anterior, occurring in the cervical and dorsal spine [69].

Only one case in the literature has associated a herniated disc at the T3/T4 level with surgery [70]. Several cord herniations have occurred at the vertebral body level rather than the intervertebral disc area [61]. Another favored explanation, proposed by Japanese authors [58], suggests that the ventral dura may be duplicated, allowing the spinal cord to herniate through the inner layer. These findings challenge earlier theories by Wortzman and Masuzawa, which posited that STSCH results from the spinal cord herniating into a preexisting ventral meningocele [71,72].

Given the late presentation of most patients (typically middle-aged adults) and the absence of spinal deformities such as spina bifida in the literature, STSCH is likely an acquired phenomenon, emerging in adulthood and invariably associated with de novo symptoms. It is crucial to recognize that the spinal cord is located ventrally within the thoracic canal, and all cases of spontaneous spinal cord herniation have been either ventral or ventrolateral. An inflammatory process involving the spinal cord and/or meninges may initiate ventral adherence of the cord to the dura due to its anatomical positioning. This initial event may be asymptomatic or cause mild clinical symptoms, but as the spinal cord becomes tethered, symptoms gradually worsen until the cord begins to herniate through a dural defect likely caused by inflammation, adhesion, and the pulsatility of the now-fixed spinal cord [58]. Adhesions are commonly found at the dural edge of the defect and may prevent the reduction of the herniated cord. Thickened arachnoid tissue has been documented by many authors [58], believed to arise from inflammation due to cord incarceration. In one case, ventral adherence of the cord occurred prior to herniation, suggesting that the inflammatory process causing arachnoid thickening begins early in the disease’s pathogenesis [58]. It is likely that the pulsations of the now-anchored spinal cord lead to erosion of the dura, resulting in a dural tear or defect. Consequently, the cord progressively herniates through this defect, with pulsating CSF seeping around the herniating cord, fostering the formation and gradual enlargement of an anterior extradural cyst. Intraoperatively, the ventral wall of the cyst was covered with a whitish membrane, a finding reported by several authors [59], indicating the presence of pseudocapsular tissue rather than dura.

The identification of a clinical and MRI spectrum within STSCH has led to the proposal of a broader term, though less commonly used in clinical practice, known as “thoracic anterior spinal cord adhesion syndrome” (TASCAS) [73]. This term describes a clinical presentation that is indistinguishable from patients with STSCH but lacks imaging or surgical evidence of an actual cord hernia, combined with MRI findings of anterior cord deviation. The authors suggest that true herniation represents the extreme end of a pathological spectrum, while other cases of anterior cord deviation and atrophy—possibly due to focal vulnerability, defects in the anterior dura, or anterior tethering of the cord—exhibit similar clinical symptoms. The association of thoracic myelopathic symptoms with an anteriorly displaced and thinned thoracic cord (with or without radiological or surgical evidence of cord herniation) has been proposed as part of the TASCAS spectrum. Ewald et al. [74] illustrated a case showing the progressive development of an anterolateral T6 cord herniation on repeated MRI studies, with associated clinical symptom progression over two years prior to surgical intervention. The authors presented a single-center series of 16 patients with clinical and radiological findings consistent with TASCAS, highlighting both typical and less common imaging appearances. A significant differential diagnosis for anterior cord adhesion is displacement caused by an arachnoid cyst or other lesions. Most lesions were identified at mid-thoracic levels (T3 to T9/10), with 63% located between T4/5 and T7/8. In all instances, the spinal cord was displaced anteriorly, with focal cord atrophy observed in 14 out of 16 cases. T2 hyperintensity within the affected cord was a common feature (13.1%) [73]. A demonstrable anterior dural defect was not frequently observed, occurring in only 3 out of 16 cases with true herniation in 4 out of 16 cases, uncertain features in 2 out of 6, and adhesion in 6 out of 16 [73]. In the proposed series, 43.7% of patients underwent posterior surgical approaches, and, among them, 71.4% were found to have significant oval defects in the anterior dura, with asymmetric herniation of the thoracic cord into the outpouching. However, only 20% of surgically treated cases demonstrated an anterior dural defect on preoperative imaging. In another 20% of cases, no definitive dural defect was identified through imaging or surgery; instead, the cord was displaced and tethered in an anterior position by adhesions from surrounding arachnoid and/or dentate ligaments [73]. Generally, the cord appeared sharply kinked anteriorly at the level of herniation, in contrast to the soft curve of cord displacement caused by a posteriorly positioned lesion. In 87.5% of cases, the cord abnormality occurred immediately adjacent to the intervertebral disc or with an intervertebral disc at the upper margin of the cord lesion. In more than half of them (57.1%) with the lesion spatially related to an intervertebral disc, discal abnormalities were noted, most commonly of a degenerative nature [73]. Progressive gait difficulties are the classic initial symptom of this syndrome [73].

The association of STSCH with other SIH symptoms has not been fully investigated. Although orthostatic headache is the most common symptom of CSF leaks, many patients report additional symptoms, and some may experience no headache at all [75]. Other symptoms associated with CSF leaks include disturbances in oculomotor and cochlea-vestibular functions, cognitive decline, and balance issues [76,77,78,79]. Furthermore, both current and past history of orthostatic headache and red blood cells in CSF have been documented in patients with SIH combined with a VLISFC [38,80,81,82,83].

The proposed causes for the development of a ventrally herniated spinal cord range from acquired to congenital factors [84,85]. Erosion of the dura due to a ruptured disk or minor trauma has been suggested as an acquired cause [26,86,87,88,89,90]. When exploring the etiology of ventral herniation of the spinal cord without an obviously predisposing event, several considerations must be taken into account [91].

(1) First, the adult spinal cord lacks growth potential unless a neoplasm is involved.
(2) Second, a spontaneous opening in the ventral dura seems improbable given its consistency and lack of movement; if movement plays a role, it occurs along the smooth connective tissue: the posterior longitudinal ligament (PLL).
(3) Third, the spinal cord is highly sensitive to trauma, and any protrusion anchored in a dural defect is likely to coincide with a severe neurological deficit.
(4) Fourth, most patients present symptoms in their sixth decade, having gradually developed signs, often following an acute temporary neurological deficit after a minor fall several years before symptom onset.
(5) Finally, biopsies or resections of the herniated segment typically do not result in neurological deficits [91].

Based on these observations, an acquired origin appears highly unlikely. Although a congenital cause is often evoked, it is rarely specified or detailed [68,92,93,94]. However, an embryological explanation for the so-called herniation of the spinal cord is plausible. During neural tube formation, neural crest cells arise at the dorsolateral aspects and spread to the ventral side [95]. During this process, mesenchymal cells from the somites mingle with neural crest cells, forming the meninx primitiva, a precursor to the meninges. By gestational days 30 to 32, the neural tube is already covered by a single cell layer representing the future pia [95]. The layer on the ventral side of the neural tube and the dorsal side of the intervertebral disk of the vertebral body, consisting of neural crest and mesenchymal cells, is reported to be thicker than the lateral or dorsal regions [95]. It can be subdivided into three layers: (1) an outer perichondral layer adjoining the vertebral body, (2) an intermediate layer that will develop into the PLL, and (3) an internal layer forming the ventral dura mater. It is hypothesized that within this thicker layer, neural crest cells accumulate and differentiate into neural tissue instead of dura, due to their role in forming spinal ganglia. Consequently, an aggregate of non-functional neuronal cells (an appendix) could be established adjacent to the spinal cord, partially covered by pia, resulting in a defect in the dura or PLL (if present), or, in rare cases, a small cavity within the vertebral body. The formation of this appendix could occur between 30 and 60 days of gestational age, depending on the studied specimens, as differentiation of the dura mater occurs prior to this gestational age [4,95,96]. This neural tissue does not contribute to any neurological function. In fact, a biopsy or resection of a portion of the spinal cord typically leads to neurological deficits. The absence of reported deficits following a biopsy suggests that the tissue was non-functional; however, some nervous cells were observed upon histological examination [62,74,97,98,99,100]. The transdural appendix may tether the spinal cord and this phenomenon may become clinically significant. A severe (temporary) neurological deficit can occur after a minor incident, with deficits potentially developing gradually with age. This hypothesis is supported by findings noted in radiological examinations: nuclear trails [101], clefts in the vertebral body at the level of MRI pathology [102,103], and cavities within the vertebral body at the level of pathology [104]. Nuclear trails or clefts indicate improper fusion of the somites. Cavities could only exist if they were present at the time of the abnormality’s development, as a spinal cord fixed in a dural opening would not erode the bone. This hypothesis also accounts for the absence of postoperative CSF leaks. Although a dural defect was consistently identified and frequently enlarged to facilitate complete dissection of the protrusion, no attempts were made to close the defect. This could only occur if the dura was firmly fixed with surrounding ligaments. It may also explain the atrophic appearance of the spinal cord at the affected level. Due to the tethering of the spinal cord, gradual circulatory changes would occur, leading to atrophy as a consequence. If this theory holds true, the term idiopathic ventral herniation of the spinal cord is a misnomer. Congenital transdural appendix of the spinal cord would be a more accurate designation, as its cause is clearly defined. This term would not refer to an active process but rather to the result of a developmental disorder: an inert segment adherent to the spinal cord.

### 3.4. Spinal Arachnoid Web

Spinal arachnoid web (SAW) was not initially classified as a “duropathy,” but recent proposals by some neurosurgical authors suggest its inclusion within this category, given that the clinical manifestations are similar to those of STSCH [105]. The pathological hallmark of arachnoid web is found on the dural side of the spinal arachnoid membrane [105,106]. In fact, SAW is characterized by an abnormal thickening of the bands of intra-dural arachnoid tissue that extend from the pial surface of the dorsal aspect of the spinal cord [107,108]. These webs are sometimes regarded as a variant of an arachnoid cyst, remnants of disrupted or collapsed arachnoid cysts, or even as incomplete formations of an arachnoid cyst [107,109,110].

SAWs may develop following a reported history of trauma, supporting the theory of a preceding arachnoid cyst. However, non-traumatic arachnoid webs were described and a congenital origin associated with a thickened ligamentum flavum has been proposed [111,112]. Notably, up to 2018, 31 cases of SAW had been documented in the literature, with only 13 confirmed at surgery [113,114], suggesting that SAW is an extremely rare entity and raising the question of whether it is truly rare or merely under-diagnosed or under-reported.

It was first described by Mallucci et al. [115] in 1997 and the most extensive series of surgically confirmed SAW patients to date was published in 2019 by Nisson et al. [116], identifying only 43 cases. A recent systematic review, after excluding cases without surgical confirmation, reported 197 cases of SAW [117]. The age range varied from 24 to 81 years (average age 55.6 years), and 61.4% of the patients were male. A history of trauma, surgery, or multiple sclerosis was noted in 9.94%, 4.42%, and 1.66% of cases, respectively. The average time from trauma/surgery to symptom onset was 31 months (range: 0.25–240 months), with 68% of patients experiencing symptom onset after 1 year [117].

The level of posterior cord indentation was described in 171 cases [117]. It was located in the cervical spine (C7) in 1 case (0.58%), in the upper thoracic spine (T1–T4) in 45% of cases, in the mid thoracic spine (T5–T8) in 52.63% of cases, and in the lower thoracic spine (T9–T12) in 1.75% of cases [117]. Syrinx or cord T2 hyperintensity was found in 75.13% of cases, extending rostrally in 62.62% of cases, caudally in 29.90%, both in 5.61%, and at the same level of indentation in 11.21% of cases [117].

Pathology revealed mostly fibrous connective tissue, which is bordered sometimes by arachnoid cells or meningothelial cells, with CD3-positive T cells found in two cases. SAW is characterized by fibroconnective adhesions [71,118,119,120], although the pathophysiology remains poorly understood. It is hypothesized that these webs may arise from segmental inflammation of the arachnoid mater or intermediate leptomeninges, potentially triggered by various factors such as trauma, surgery, hemorrhage, or infection [121,122]. Additionally, a history of systemic inflammation, the use of intrathecal pain pumps, or conditions like myelitis (including multiple sclerosis or transverse myelitis) may also contribute to their formation [116,123,124]. There is a suggestion that SAW could develop secondary to ruptured arachnoid cysts [110,123,124], as illustrated in a case report where a thickened arachnoid membrane was observed expanding like a septum within an adjacent arachnoid cyst [125]. Delgardo et al. [106] presented the largest series of histological findings of SAW, reporting that, in all 16 cases examined, fibrous connective tissue was found, often associated with meningothelial cells (ten cases), arachnoid cells (one case), and calcifications (three cases). Some studies have noted a small number of infiltrating CD3-positive T cells, indicating an inflammatory process [122,126].

SAW is frequently associated with syringomyelia. The origin of syringomyelia associated with SAW has not been fully clarified, with several hypotheses proposed. One theory suggests a valvular mechanism caused by CSF blockage by the arachnoid membrane, leading to increased proximal pulse pressure and the subsequent passage of CSF into the spinal cord through perivascular spaces [116,127]. Other theories propose a significant drop in subarachnoid space pressure distal to the blockage, generating a gradient between the ependymal duct and the space, which explains the distal presence of the syrinx. These theories resemble the piston theory or hydrodynamic drive described in Chiari malformation syringomyelia, as well as the Venturi suction effect caused by pressure imbalances on either side of the blockage [112,116,127]. The most convincing theory posits that compression exerted by the arachnoid web disrupts the transmission of the systolic pulse pressure wave of the intramedullary CSF, whether caudally, cranially, or both. This disruption results in a pressure differential originating from the center of the cord, where pressure is elevated, extending outward and leading to dilation of the spinal cord. This dilation increases extracellular space and allows for CSF accumulation within the cavity. Partial obstruction may also facilitate spinal cord dilation through a suction effect or reduction in pressure, which could elevate the velocity of CSF in the narrowed segment [108,128].

Clinical signs associated with SAW are related to cord compression, with patients commonly reporting sensory disturbances, followed by pain, weakness, gait abnormalities, and sphincter dysfunction [117]. The clinical signs were predominantly sensory disturbances (68.50%), pain (64.64%), and motor weakness (60.22%), including four cases with fine motor hand difficulties [117]. Additionally, gait disturbances were present in 52.48% of cases, including three cases of intermittent claudication. Sphincter disturbances were noted in 22.10% of cases, hyperreflexia in 25.97%, and hyporeflexia in 3.31% [117]. Symptoms were localized to the lower limbs in 69.61% of cases, the trunk in 37.56%, and the upper limbs in 25.41%. Among patients with upper limb symptoms, 51.85% had a syrinx extending to the cervical spine [117]. Symptoms in the lower limbs are the most frequently involved (69.61%), likely due to the high incidence of thoracic SAW locations, followed by symptoms in the trunk and upper limbs. A trend was noted concerning patients presenting with upper limb symptoms; a significant proportion (51.85%) had a syrinx extending up to the cervical spine [117]. Conversely, Laxpati et al. [129] observed that more superiorly located arachnoid webs were more frequently associated with upper extremity symptoms. The mechanisms underlying upper limb symptoms in cases of SAW without cervical syrinx remain unclear; however, they may stem from subtle variations in CSF flow dynamics within the ependymal canal above the SAW, potentially leading to mild compression of the medial aspects of the spinothalamic and corticospinal tracts.

Despite SAWs being thought to share a common pathophysiology with arachnoid cysts [107], the mechanisms culminating in the formation of an arachnoid web remain largely unknown. Several theories have been proposed, including forceful CSF flow resulting in arachnoid herniation into a congenital dural defect, as well as post-traumatic, post-infectious, and post-surgical etiologies [130,131,132,133]. Consideration is also being given to an idiopathic form of thoracic SAW [113]. Thoracic SAWs are more commonly located dorsal to the spinal cord and have a predilection for the upper thoracic segment of the spinal cord [107]. To date, there is no explanation for this segmental localization of the web [107]. Like the webs, available information regarding the associated syrinx is limited. The location of the syrinx varies in relation to the associated thoracic SAW, as it may be caudal but occurs more commonly rostral to the web itself [107]. The syrinx’s location relative to the arachnoid web is believed to be in the area where the intramedullary pulse pressure is lower compared to the opposite side of the web [131]. Greitz introduced the “Venturi effect” as a probable explanation for the formation of a syrinx, stating that the arachnoid web interrupts the transmission of systolic pulse pressure to the distal CSF, thereby altering intramedullary pulse pressure. This creates a pressure gradient from the center of the cord outwards, resulting in cavitation within the spinal cord [134]. This “suction effect” theory challenges the prevailing notion that increased pulse pressure in the subarachnoid space forces CSF through the spinal cord into the syrinx. Patients with thoracic SAW often present with neuropathic back pain, features of compressive myelopathy, or radiculopathy. Symptoms may include episodic lower extremity weakness, sensory disturbances, and bowel and bladder incontinence. Clinical examination may reveal hyperreflexia, spastic paraparesis, clonus, and gait instability [113,135]. The patient history may also indicate an antecedent surgery, infection, or trauma, although this is not always present. There is an almost 2:1 female-to-male predominance, with ages ranging from the 4th to the 7th decade [107].

### 3.5. Superficial Siderosis

As previously mentioned, SS of the CNS is characterized by the presence of hemosiderin deposits on the surfaces of the brain and spinal cord [18,19,20]. This deposition results from recurrent and persistent bleeding into the subarachnoid space, with a notable affinity for the cerebellum, spinal cord, and cranial nerves I, II, and VIII. Patients typically present in adulthood with gradually worsening ataxia, primarily affecting gait (and less frequently, appendicular movements), along with sensorineural hearing loss, likely due to prolonged exposure of the long glial segment of the eighth cranial nerve to CSF [20]. The clinical triad associated with infratentorial SS includes gradually worsening hearing loss, ataxia, and myelopathy. The preferential involvement of the vermis may explain why gait ataxia is more common than appendicular ataxia, potentially leading to misdiagnosis as degenerative cerebellar ataxia. The progressive sensorineural hearing loss often has both retrocochlear and cochlear components, typically accompanied by tinnitus affecting high frequencies, and is generally bilateral but may be asymmetric [136]. Hearing loss might also involve vestibular failure, which can be a rare initial symptom [137]. A medical history indicating low-pressure headaches can serve as a clue to a dural tear as a likely cause of SS [138]. Cognitive impairment, often manifesting as executive dysfunction, may be present but is frequently overlooked, likely due to hemosiderin deposition in the medial and inferior frontal cortex [139].

Approximately half of the patients with this condition have a spinal dural defect, where chronic hemorrhage at the leak site is likely responsible for the development of SS [18,19,20]. A study in patients with SIH and VLISFC indicated that long-term follow-up revealed two-thirds of patients with SS remained asymptomatic and MRI findings suggested a limited burden of siderosis [17]. It remains uncertain whether these patients would have developed symptoms if the spinal CSF leak had gone untreated; however, serial imaging displayed progressive SS, indicating ongoing subarachnoid bleeding until surgical repair of the CSF leak was performed. The primary objective of surgically repairing the underlying spinal CSF leak is to halt the progression of SS, and if surgery is timely performed, the condition can be reversible [17].

Patients with SS often have a history of trauma or prior intradural surgery as common risk factors [1,2,18,19,20,21]. Remote trauma is frequently presumed to be the cause of a dural tear associated with a VLISFC [1,2]. Dural tears have also been linked to protruding discs, osteophytes, or a combination of these factors, along with calcifications between the dura and arachnoid [1,2,21]. In some instances, a herniated thoracic disc without an apparent dural defect has been suspected of causing SS in patients with VLISFC [140]. Some researchers have noted the presence of “venous tissue,” “venous adhesions,” or “venous anomalies” in relation to the dural defect [39]. The ventral location of the fluid collections suggests that degenerative disc disease or osteophytes may contribute to the dural defect, even when these issues are not clearly visible on imaging or during surgery. A preference for VLISFC has not been documented in craniospinal hypovolemia. In one case of SS with pachymeningeal enhancement and VLISFC, a dural defect adjacent to a calcified disc protrusion was identified on a dynamic CT myelogram but was not observed during surgery [141]. The authors proposed that the defect might have been small or that the leak was intermittent and positional. In that case, a fat graft was placed in the epidural space, and a sealant was injected, leading to radiologic resolution of the meningocele and pachymeningeal enhancement, along with the resolution of CSF xanthochromia [141]. Another possible cause of dural defects and CSF leaks may involve areas of preexisting dural hypoplasia, often located near root sleeves. This phenomenon has been well documented in SIH and may relate to connective tissue matrix disorders.

SS typically arises from chronic low-grade bleeding into the subarachnoid space [1,21]. Hemoglobin from red blood cells in the CSF degrades into globin and neurotoxic heme [20,65,142]. In response to neurotoxic heme, neuroglial cells produce heme oxygenase and apoferritin. Heme oxygenase converts heme into free iron and biliverdin, while apoferritin binds free iron to create ferritin, which eventually forms hemosiderin. This process is likely neuroprotective, mitigating neuronal damage from free iron. However, the continued presence of blood in the subarachnoid space can exceed the capacity for ferritin production, leading to unbound iron and subsequent free-radical-induced neuronal injury. Hemosiderin accumulates in brain or spinal cord areas adjacent to the CSF, resulting in a characteristic marginal and confluent T2-hypointensity on MRI, known as SS, which is most reliably seen on iron-sensitive sequences. The preferential involvement of posterior fossa structures, especially the cerebellum, is likely due to increased ferritin synthesis by the abundant Bergmann glia present there. Furthermore, CSF flow studies indicate that the posterior fossa structures are among the first to be covered; thus, continuous contact with hemorrhagic CSF might play a more critical role in the disorder’s pathogenesis than stagnant fluid exposure.

Typically, SS is a very slowly progressive disorder. If a source of bleeding is identified and surgically addressed, some patients may experience clinical improvement. However, predicting which patients will benefit from surgery is challenging, and the primary goal of surgical intervention should be to prevent further progression by removing the source of chronic bleeding [17]. In cases of long-standing SS, the benefits of removing the bleeding source may be less clear, and some authors express concerns that the disease may continue to progress despite surgical correction [17]. Patients with chronic SS often suffer from irreversible neural tissue damage, which may limit the potential benefits of any intervention. Some authors suggest that continued progression despite therapy could be related to long-standing siderosis initiating an irreversible neurodegenerative cascade, leading to the failure of reparative mechanisms after reaching a “point of no return” [4]. This underscores the necessity for early intervention, especially in cases of SS with identifiable active bleeding [4]. Functionally relevant clinical improvements are considered the most meaningful outcomes. Neuronal injury in SS is caused by unbound iron, which occurs when microglial cells’ capacity to biosynthesize ferritin and hemosiderin is overwhelmed.

Along with SS, dural defects may lead to symptoms of SIH from CSF leaks or STSCH, within the spectrum of duropathies [1,143]. Although there may be a medical history of SIH, ongoing clinical symptoms are typically absent [1,2,18,19,20,21,144,145]. This likely relates to compensatory mechanisms due to the chronicity of the process associated with SS. Myelopathic presentations may arise from cord herniation or dynamic cord compression related to intraspinal fluid collections [143]. While SS generally spares the nerve roots, rare clinical or electrophysiological evidence of lower motor neuron involvement may occur, potentially due to arachnoiditis from blood products in the CSF or from motor polyradiculopathy related to stretching of ventral nerve roots caused by a VLISFC [143]. In some instances, lower motor neuron signs such as bibrachial amyotrophy and fasciculations may present alongside preserved or brisk reflexes, potentially leading to consideration of motor neuron disease. However, SS does not directly affect the peripheral nervous system. This is because the conversion of heme to ferritin and hemosiderin necessitates microglia and astrocytes, a process that does not occur in the Schwann cells of the peripheral nervous system [59,142,146,147]. Therefore, while hemosiderin may offer some protective effects against neuronal injury, a decrease in MRI evidence of hemosiderin over time may not serve as a reliable parameter for serial monitoring. Imaging acquisition parameters can also influence the appearance of hemosiderin, which should be taken into account when making comparisons [4,17]. Reliable indicators of successful surgical repair include the resolution of CSF xanthochromia or elevated red blood cell counts, as well as the resolution of intraspinal fluid collections in cases of dural tears [80,81,82,83].

## 4. Neuroimaging Patterns

As previously anticipated, the neuroimaging patterns of duropathies are variable and recognize some common features.

### 4.1. Dural Tear

The cause of SIH is a spontaneous CSF leak occurring at the spinal level [78]. As previously anticipated, at least three distinct types of spontaneous spinal CSF leaks were identified [10]. Type 1 CSF leaks arise from a dural tear either ventral to the spinal cord (type 1a) or (postero)lateral to the spinal cord (type 1b). Type 2 CSF leaks are associated with meningeal diverticula, classified as simple (type 2a) or complex (type 2b, involving dural ectasia). Type 3 CSF leaks are characterized by the presence of CSF-venous fistulas.

For patients with a high clinical suspicion of SIH, the initial imaging evaluation begins with a comprehensive assessment of the neuraxis, including a brain MRI and a total spine MRI. The brain MRI is evaluated for signs of intracranial hypotension, while the spine MRI aims to identify VLISFC or the source of the leak. A typical routine brain MRI protocol to assess the intracranial sequelae of SIH includes sagittal T1-weighted non-contrast, T2-weighted, T2-Fluid Attenuated Inversion Recovery (FLAIR), diffusion-weighted imaging (DWI), susceptibility-weighted imaging (SWI), and contrast-enhanced 3D T1-weighted imaging [148]. The intracranial findings may include pachymeningeal enhancement, distension of the dural venous sinuses, a sagging brainstem, SS, enlargement of the pituitary gland with reduced size of the suprasellar cistern, subdural effusions and/or hematomas, decreased mamillo-pontine distance, reduced prepontine cistern distance, decreased ponto-mesencephalic angle, and diminished interpeduncular angle. Some of these features are incorporated into the Bern score, which aims to define the likelihood of detecting a CSF leak of any type on CT myelography [138]. The presence of any of these imaging findings increases the pretest probability of identifying positive findings on a spine MRI in patients with SIH, thus warranting further investigation for spinal CSF leaks or CSF-venous fistulas. Most CSF leaks leading to SIH occur in the spine, so an evaluation for patients with high clinical suspicion of SIH must include imaging of the entire spine. High-resolution isovolumetric 3D heavily T2-weighted fat-saturated imaging is essential for identifying extradural CSF collections. This imaging technique, available under various vendor names such as sampling perfection with application-optimized contrasts by using different flip angle evolution (SPACE sequence; Siemens), SPINEVIEW (Philips), and CUBE (GE Healthcare), provides greater spatial resolution, minimizes CSF pulsation artifacts, and can be reconstructed into multiple planes to better visualize small and large extradural CSF collections that may be difficult to detect on routine 2D T2-weighted images. The 3D heavily T2-weighted fat-saturated sequence is also effective in identifying meningeal diverticula, which can serve as a source of CSF leaks or CSF-venous fistulas [148,149]. If a spinal longitudinal extradural collection positive is detected on the spine MRI, the next step is to perform dynamic CT myelography to locate the leak site. The dynamic CT myelography is beneficial for pinpointing the exact location of the leak. DSM may also be helpful, particularly for identifying large tears or rapid leaks due to its higher temporal resolution and lower radiation dose; however, it can be more susceptible to motion artifacts [150]. A prone DSM may struggle to identify a ventral leak near the shoulders (C7–T3) because of overlapping anatomical structures that obscure the ventral leak site at this level. In dynamic CT myelography, both lumbar puncture and myelography are performed with the patient positioned on the CT scanner, and the patient is rapidly scanned following the administration of intrathecal contrast. The positioning of the patient depends on the location of the extradural fluid collection or the suspected type of leak [148,149]. If no extradural fluid collection is identified, the patient will proceed to conventional CT myelography [151,152]. Should the findings from conventional CT myelography be negative, bilateral lateral decubitus DSM may be performed to search for CSF-venous fistulas, dural tears, or meningeal diverticulum leaks. Additionally, decubitus CT myelography may be considered, particularly with the increasing availability of photon-counting detector CT, which allows for higher spatial resolution and reduced noise [149,153].

**Figure 2:**
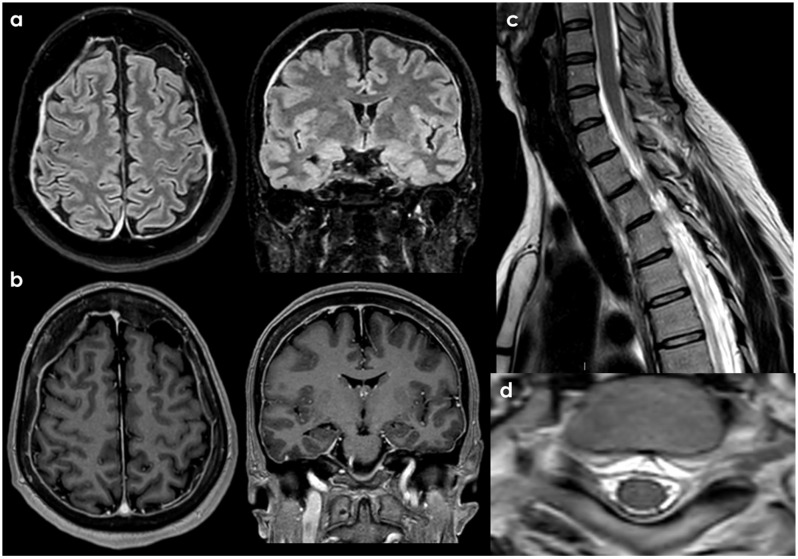
Brain and spine MRI findings in a patient with SIH (**a**,**b** and **c**,**d**, respectively). In (**a**), axial and coronal T2 Fluid Attenuated Inversion Recovery (FLAIR) images are proposed with a spontaneous pachymeningeal hyperintensity with contrast enhancement in the corresponding post-contrast T1W images (**b**). In the sagittal and axial T2W spine MRI (**c** and **d**, respectively), a VLISFC is evident. SIH: spontaneous intracranial hypotension; VLISFC: ventral longitudinal intraspinal fluid collection.

An example of neuroimaging findings in a patient with SIH is proposed in Figure 2. It should be considered that most patients with SIH have only a few of the classical neuroimaging findings on brain MRI, with 80% of patients having at least one finding [15], and the most frequent one is diffuse pachymeningeal enhancement (73–83%) [15]. As for clinical signs and for neuroimaging findings, there is a well-established time-dependency, and in long-standing disease, the imaging pattern may be subtle.

**Figure 3:**
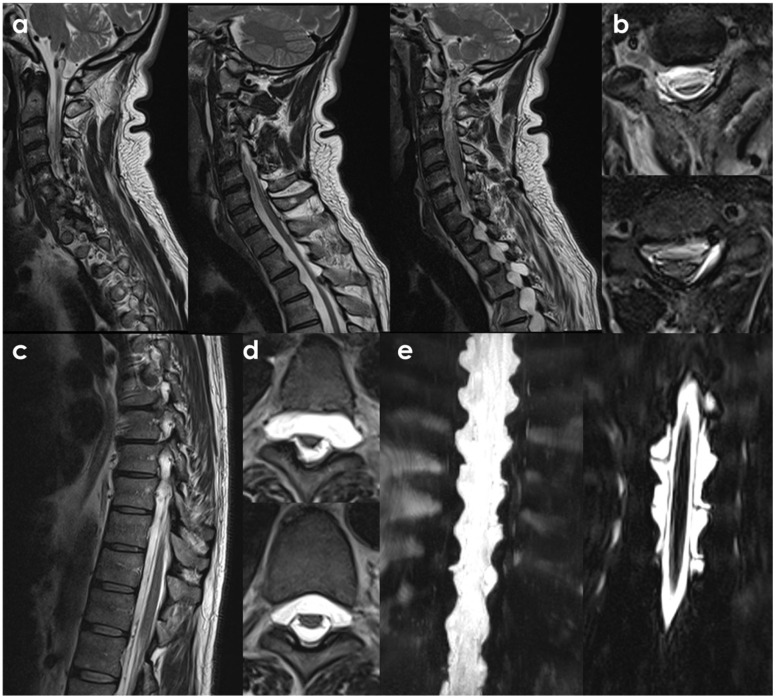
Spine MRI of a patient with brachial amyotrophy with a progressive course within few years after a major trauma with brachial plexus roots avulsion on the right side about 20 years before. Panel (**a**,**b**) are sagittal and axial (cervical level) T2W images, showing VLISFC and markedly reduced section of the spinal cord. Panel (**c**,**d**) show the corresponding sequences and findings in the dorsal segment. Panel (**e**) shows MyeloMRI with extensive VLISFC. SIH: spontaneous intracranial hypotension; VLISFC: ventral longitudinal intraspinal fluid collection.

Sometimes, a post-traumatic etiology can be strongly supposed, as in the case illustrated in Figure 3.

### 4.2. Superficial Siderosis and VLISFC

**Figure 4:**
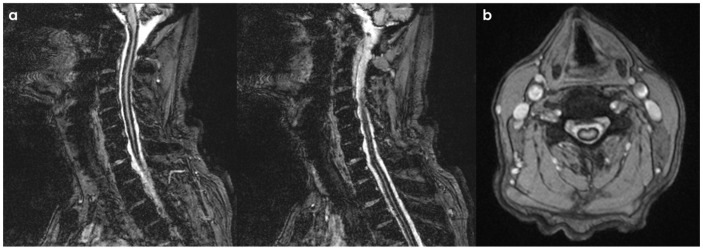
T2W sagittal (**a**) and axial (**b**) MRI at the cervical level with extensive circumferential hypointense lining of brainstem and spinal cord, suggestive for siderosis.

**Figure 5:**
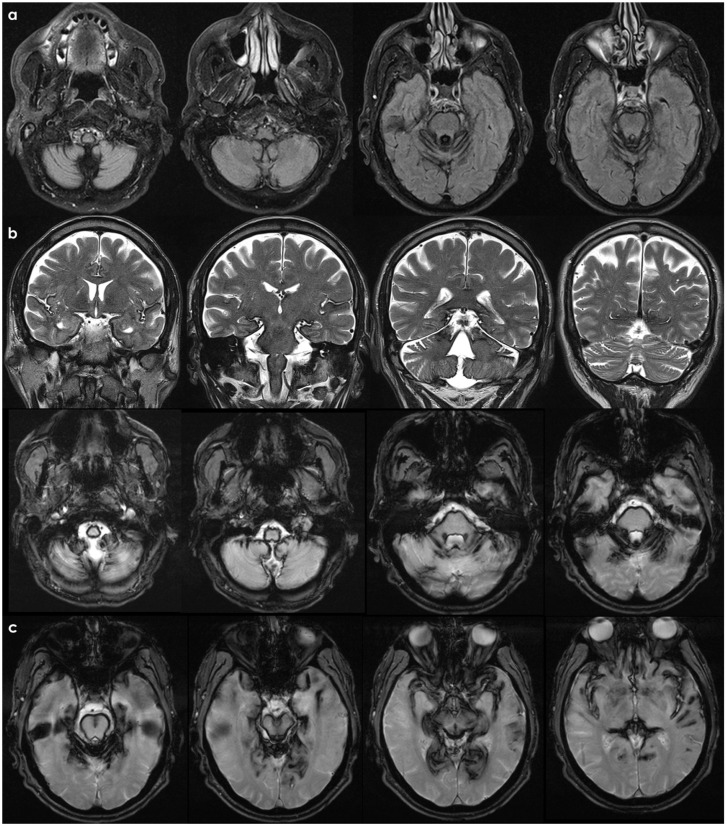
Brain MRI in the same patient of Figure 3, showing extensive, mainly infratentorial siderosis, well evident in axial FLAIR (**a**), coronal T2W (**b**) and axial T2* (**c**, two lines).

SS of the CNS is characterized by the deposition of hemosiderin in the subpial layers of the brain and spinal cord [1,2]. A history of prior injury or intradural surgery—often involving the posterior fossa—is commonly noted. There may be a significant time gap, sometimes spanning decades, between the initial triggering event and the onset of symptoms related to SS. MRI is the preferred diagnostic tool, with the hallmark finding being a distinctive, marginal, confluent T2 hypointensity observed on the surface of the brain and spinal cord [2]. This finding is particularly evident on gradient-echo sequences. As MRI technology has advanced, asymptomatic cases of SS have been identified, complicating efforts to ascertain the true incidence of the condition. Additionally, SS has recently been recognized as an MRI indicator of cerebral amyloid angiopathy [152]. In CSF analyses, the presence of red blood cells or xanthochromia may be noted. Despite thorough evaluations—including brain and spine MRI, CT myelography, MR angiography, and cerebrospinal angiography—the source of the bleeding frequently remains elusive. An example is illustrated in Figure 4 and Figure 5.

VLISFC is frequently associated with SS, SIH, bibrachial amyotrophy, and STSCH [2,23,38,154,155,156,157]. A common feature among these conditions is the presence of a dural defect. Typical sources of recurrent bleeding in SS often include neoplasms, vascular malformations, and various dural pathologies, such as root avulsion or intraspinal cysts (meningoceles or pseudomeningoceles) [1,2]. A single-center study on 30 patients with SS found that a VLISFC was observable on MRI in 14 out of 30 cases, usually extensive. Other studies confirmed this finding [1,2,18,19,20,21]. The classification of these fluid-filled collections has varied, with terms such as intradural, subdural, extradural, extra-arachnoid, extrathecal, or indeterminate being employed. They are often referred to as meningoceles, pseudomeningoceles, epidural cysts, arachnoid cysts, or simply CSF loculations or “collections”. The high prevalence of VLISFC suggests that the associated dural defect may play a significant role in the pathogenesis of SS, as in SIH. This condition is often accompanied by extra-arachnoid fluid collections, both supratentorially (over the cerebral convexities) and at the spinal level (ventral, dorsal, or circumferential longitudinal CSF collections linked to the spinal subarachnoid space) [158,159,160,161]. Classic neuroimaging findings associated with SIH, such as pachymeningeal enhancement, foreshortening of the suprasellar cistern, sagging of the midbrain, and flattening of the ventral pons, have also been observed in some SS patients with a VLISFC [83,141,146,147]. In one case, the low CSF opening pressure and pachymeningeal enhancement in a patient with SS and a VLISFC improved following the repair of the dural defect, which was also accompanied by resolution of the VLISFC, CSF xanthochromia, and an increase in red blood cell counts [141]. Additionally, some patients with SS and a VLISFC may exhibit neuroimaging signs of increased pial vascularity [38,83,141,162]. Increased vascularity has also been noted in neuroimaging of patients with SIH [160,161]. It has been suggested that pial siderosis could lead to sclerosis of epidural veins, resulting in venous hypertension and prominent vascularity [141,163]. The pial vascularity observed in certain SS patients with a VLISFC has been shown to resolve following the repair of the dural defect, indicating a connection to SIH and vascular engorgement rather than pial siderosis causing sclerosis of the epidural venous plexus [141]. This distinction is critical, as the vascular engorgement seen on imaging in SS patients may prompt unnecessary angiography in search of a bleeding source, which is often not present [65,163]. As previously noted, among the MRI findings of an unrelated condition, Hirayama disease, there is forward displacement of the posterior dura during neck flexion, which occurs due to the enlargement of the posterior epidural space, likely resulting from engorgement of the epidural venous plexus [52,164,165,166]. This condition ultimately leads to compression of the ventral cord.

Less frequently, SS can result from isolated episodes of subarachnoid bleeding caused by vascular malformations, CNS tumors, craniospinal surgery, or CNS trauma. This subgroup typically does not present with ataxia or hearing loss, and the hemosiderin deposition is primarily localized around the site of bleeding rather than in the posterior fossa. This group is referred to as type 2 or secondary SS [20]. Some authors have proposed the terms classical-type SS and localized-type SS [167].

Dural defects, trauma, and craniospinal surgery are commonly identified causes of SS [1,2,20,168]. A Mayo Clinic study involving 30 SS cases identified MRI-detected dural abnormalities that could explain chronic subarachnoid bleeding in 18 patients [1,2]. Similarly, a recent series of 65 SS cases from the National Hospital for Neurology and Neurosurgery in the UK found dural abnormalities in 40 patients [20,21]. These abnormalities may include dural tears resulting from disc herniations, intrinsic dural diseases linked to connective tissue disorders, traumatic nerve root avulsions, or postoperative pseudomeningoceles. Dural tears are often associated with VLISFC, as in SIH without SS [20]. As previously proposed, the ventral positioning of these fluid collections is likely due to a disc herniation (often calcified) or an osteophyte (which can be spiculated) causing the dural tear [1,2,18,19,20]. It is important to note that the herniation or osteophyte may not be present during surgical exploration. The presence of a “nuclear trail sign” outlining the path of a migrated disc may provide indirect evidence linking it to the dural tear [101]. The dura is most closely attached to the dorsal surfaces of the vertebral bodies and intervertebral discs from C5 to T7, where high-flow CSF leaks are most frequently observed [101].

Dural tears can also result from trauma, with root avulsions sometimes observed in brachial plexus injuries. Pseudomeningoceles, whether intracranial or spinal, may arise from trauma, often surgical [144,169,170,171,172,173,174,175,176]. Dural ectasia may be present in some patients with SS, even without an obvious dural defect, as seen in conditions such as Marfan syndrome, neurofibromatosis, and ankylosing spondylitis [20,177]. It is likely that earlier reports of idiopathic SS may have included patients with unrecognized spinal dural defects, as comprehensive imaging of the entire neuraxis was not routinely performed during SS evaluations.

Limited pathological data on patients with SS and VLISFC suggest that while the collections appear epidural, they may in some cases be intradural, resulting from CSF dissection through a partial ventral dural tear into an intradural space [143,176,177]. Similar findings have been observed during perioperative endoscopy, where fragile and bleeding bridging veins with trabeculae have been noted between the dural layers [178,179]. Exudation of blood from these engorged or damaged epidural or intradural vessels is speculated to be a potential source of chronic bleeding leading to SS [50,160,177]. Reports of subarachnoid hemorrhage in patients with SIH due to dural tears further support the association between SS and CSF leaks from dural defects [177,180]. In patients with SIH resulting from dural tears, red blood cells can be detected, and engorgement of the epidural venous plexus has been suspected to contribute to the CSF being xanthochromic or even blood-tinged in some instances [1]. It has also been suggested that in cases where SS related to a dural defect is associated with SIH and brain sagging, recurrent bleeding from superior cerebellar bridging veins that are stretched due to brain sagging may lead to the development of SS [1]. Tumors and vascular malformations have also been linked to SS [181,182,183]. When imaging of the neuraxis reveals a cranial or spinal tumor, a causal relationship is plausible; however, certain tumors, such as meningiomas or pituitary adenomas, could be incidental findings. In patients who have undergone surgery, SS may result from prior bleeding from the tumor or ongoing bleeding from residual tumor tissue or a postsurgical cavity. Besides tumors, subarachnoid bleeding can result from trauma or surgery, as well as from vascular malformations, although the latter are often incidental when identified during SS evaluations. While imaging may show hemosiderin deposition following isolated subarachnoid bleeding episodes, the classic clinical and imaging presentation of SS is rare [184,185]. Some patients who have received radiotherapy post-tumor resection may develop SS, as telangiectasia and cavernous angiomas can appear in the brain and spinal cord after radiotherapy, potentially serving as sources of chronic subarachnoid hemorrhage [186].

With the widespread adoption of MRI, particularly with iron-sensitive sequences like gradient-echo imaging (GRE) and susceptibility-weighted imaging (SWI), SS is increasingly diagnosed, sometimes even in asymptomatic individuals. In fact, mainly based on studies comparing imaging techniques for cerebral amyloid angiopathy, SWI is generally more sensitive than GRE-T2 for detecting cortical SS, particularly in identifying the extent and multifocality of the condition [187]. While both sequences are effective, SWI demonstrates a higher sensitivity, especially for disseminated (more than 3 sulci) cSS, detecting it in 50% of cases compared to 37.04% with GRE-T2* [187,188]. Unfortunately, no direct comparison between the two sequences is available for SS and for the evaluation of spinal siderosis.

#### Diagnostic Pathway of SS

MRI of the entire neuraxis is the preferred diagnostic investigation for SS [1,2,23]. It is advisable to perform brain MRI with contrast, as pachymeningeal enhancement—though uncommon—may indicate an underlying dural tear and associated CSF leak as the source of SS. Additionally, post-contrast studies enhance the identification rates for alternative etiologies of SS, such as tumors or vascular malformations. T2-hypointensity typically affects the surfaces of the cerebellum and brainstem but may also extend to cortical sulci and the Sylvian or interhemispheric fissures. While MRI findings in SS are distinctive and pathognomonic, they are often overlooked due to the symmetric and confluent GRE-T2 and SWI hypointensity that follows the contours of the brain and spinal cord. These findings may also be missed without iron-sensitive sequences. Cerebellar atrophy is frequently observed, particularly affecting the superior vermis and anterior cerebellar hemispheres. T2-hypointensity may also be present around the spinal cord, which may exhibit associated atrophy. Clumping or peripheralization of nerve roots due to arachnoiditis is another notable imaging feature. Dural pathology, usually manifesting as a dural tear, is the most common underlying cause of SS [1,2,18,19,20,21]. Patients with SS often present with VLISFC that indicate CSF leaks. A ventral leak may suggest a calcified disc herniation as a potential cause of the dural tear [1,2]. Conventional MRI can show VLISFC but often fails to identify the specific site of the associated dural defect. High-resolution constructive interference in steady-state (CISS) reverse MRI enhances the contrast between the dura and CSF, potentially facilitating better detection of dural defects than standard T2-weighted MRI [139,178,189]. It is important to note that the center of the fluid collection typically does not align with the location of the dural tear [139].

Therefore, additional tests aimed at identifying the dural defect, such as CT myelography (often dynamic), and less commonly, techniques like dynamic spine imaging (DSM) [1,2,3,18,19,20,21,78] or MR myelography, are necessary. Positive-pressure application can enhance the efficacy of MR myelography, as can positive-pressure application during CT myelography. A recent report combined a balanced steady-state free precession (SSFP) sequence for high resolution with a dynamic improved motion-sensitized driven-equilibrium (iMSDE) SSFP sequence to visualize motion through the dural defect for noninvasive localization and subsequent repair via a minimally invasive approach [190,191].

The choice of the appropriate technique generally depends on the available expertise at each institution. A CT myelogram can indicate a leak or suggest its presence by filling the intraspinal fluid collection. Confirming free communication of the intraspinal fluid collection with the subarachnoid space implies the existence of a dural defect. Dynamic CT myelography is preferred when a longitudinally extensive VLISFC is present [1,2,3,18,19,20,21,78,190,191,192,193]. This technique employs a multi-slice CT scanner with active scanning during contrast injection. In this scenario, the patient is positioned prone with their hips elevated above the cervical spine to facilitate gravity-assisted cephalad flow and ventral contrast accumulation during scanning [194,195]. Standard myelograms can obscure the initial site of leakage due to diffuse epidural contrast spillage, which is essential for guiding effective surgical repair of the dural tear. SS is generally not caused by macrovascular pathology. Consequently, vascular imaging of the brain or spine—including CT angiography (CTA), MR angiography (MRA), and conventional catheter angiography—typically yields no significant findings [21]. When vascular abnormalities, such as small aneurysms or venous angiomas, are identified, they are often incidental findings. Although cavernous angiomas have been associated with SS, they are usually angiographically occult and do not present the classic clinical features of SS. As a result, these angiographic tests are no longer routine in the evaluation of patients with SS, although they may still be relevant for select cases.

### 4.3. Intradural Pseudocyst

In addition to siderosis and VLISFC associated with spinal dural defects [1,2,18,19,20,21,171], another manifestation of SS is the presence of intradural pseudocysts, which can have the same imaging features of VLISFC and represent mainly an intraoperative or pathological diagnosis [105]. In fact, recent pathological examinations of the spinal dura mater obtained from SS patients with intradural fluid collections have provided insights into possible mechanisms.

The proposed mechanism for the formation of intradural pseudocysts and the persistence of subarachnoid hemorrhage involves several stages [105]:

1. A defect may develop in the inner layer of the dura mater.
2. The arachnoid membrane (AM) herniates into this defect, resulting in the development of a granulomatous lesion around the defect, which may ultimately lead to the breakdown of the herniated AM.
3. Continuous bleeding from the granulomatous tissue can result in subarachnoid hemorrhage, contributing to the onset of SS.
4. The influx of CSF through the defect may prompt the formation of an intradural pseudocyst, which can impact blood vessels in the dura mater, leading to hemosiderin deposition.

These findings suggest that the intradural fluid collection seen in patients with SS can be in some cases an intradural pseudocyst created by the dissection of the spinal dura mater. The low levels of hemosiderin pigments in the inner wall of the pseudocyst, along with the reduction in pseudocyst volume after surgical repair, indicate that the fluid within the pseudocyst contains minimal blood components and is primarily maintained by CSF influx from the subarachnoid space through the defect [105].

The upper thoracic region is particularly vulnerable to the formation of dural defects [21]. Imaging studies show that this area is the most immobile and kyphotic in the spine, suggesting that the ventral dura mater may be in close proximity to or in contact with the dorsal surfaces of the vertebral bodies or intervertebral discs during movement [194]. Excessive spinal movement, such as that caused by traffic accidents, may place significant tension on the thoracic dura mater, potentially resulting in a defect in the inner dural layer. Granulomatous lesions may then develop around this defect, leading to the destruction of the invaginated arachnoid membrane. Repeated bleeding from the granulomatous tissue can cause spinal subarachnoid hemorrhage, contributing to the development of SS in the CNS. Concurrently, CSF influx from the subarachnoid space into the dura mater through the defect can lead to the formation of an intradural pseudocyst. Hemorrhages from the granulomatous lesion may partly contribute to the pseudocyst’s formation, and the gradual expansion of the pseudocyst can affect the blood vessels in the dura mater, resulting in hemorrhage and subsequent hemosiderin deposition in the dissected dural layer.

### 4.4. Spontaneous Transdural Spinal Cord Herniation

STSCH occurs at locations where there is dural thinning or defects, allowing the spinal cord and its covering arachnoid to become incarcerated at the site of the dural abnormality. The natural curvature of the spine typically causes the spinal cord to contact the anterior surface of the dura between T2 and T8, making this region the most common site for spinal cord herniation [196,197,198,199]. CSF pulsations can exacerbate the herniation, potentially leading to erosion of the adjacent bone [200].

The typical presentation of spinal cord herniation is progressive myelopathy, which may also manifest as CSF hypotension syndrome due to CSF leakage [201]. VLIFSC, often referred to as arachnoid or epidural cysts, have been reported in association with dural defects and STSCH [109,156]. In one case, a VLISFC was observed alongside SS in a patient with STSCH [202]. However, when focal spinal cord displacement is identified along with a widened CSF space on imaging, diagnosing the underlying cause can be quite challenging. STSCH is considered a rare condition [62]. Other potential conditions that may present similarly include space-occupying CSF-isointense intradural or intraspinal extramedullary lesions, such as epidermoid cysts, intradural arachnoid cysts, spinal epidural abscesses (SEAs), cystic nerve sheath tumors, meningoceles, teratomas, synovial cysts, and epidural hematomas [62].

Frequently, the patient’s clinical history and physical examination findings are nonspecific [203]. This nonspecificity can complicate the diagnostic process and delay appropriate treatment. The main differential diagnoses on neuroimaging are reported in Table 1.

**Figure 6:**
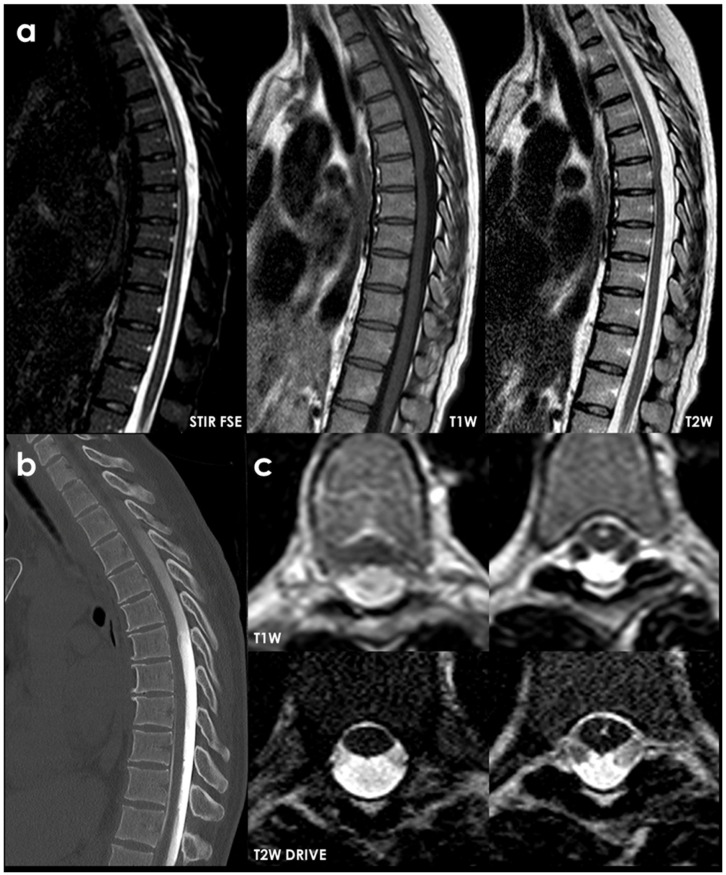
Spinal cord herniation in sagittal dorsal MRI (**a**) with the focal ventral attraction of the spinal cord at a vertebral body level, in the corresponding sagittal CT myelography (**b**) confirming the lack of contrast at the ventral side of the spinal cord at the level of attraction, and, finally, axial MRI (**c**) at the attraction level (**left** images) and under just this level (**right** images).

STSCH typically occurs between the T3 and T7 vertebrae, likely due to the natural curvature of the thoracic spine and the anterior positioning of the spinal cord in these regions. The defect usually arises at the level of an intervertebral disk, although it can also occur at the vertebral body level. In rare cases, the defect may span multiple vertebral segments [94]. Imaging findings associated with STSCH may not be specific. Both MRI and myelography typically show obliteration of the CSF space ventral to the cord, accompanied by a widened dorsal CSF space, generally without any solid or cystic mass located posterior to the cord [66]. Differentiating between focal STSCH and CSF flow artifact can be particularly challenging, as both conditions may present similar hypointensity on standard T2-weighted MR images [64]. A continuous normal CSF pulsation artifact in the widened CSF space serves as a diagnostic indicator suggesting unimpeded CSF flow, which argues against the presence of an obstructing lesion. The use of intravenous contrast during imaging is often necessary and it can help refine the differential diagnosis. STSCH typically does not enhance, while the presence of enhancement indicates a space-occupying lesion [64,204]. This distinction is critical for accurate diagnosis and appropriate management of this condition. An example of STSCH is illustrated in Figure 6.

Technological advancements, such as phase-contrast and thin-section MRI, have significantly enhanced the diagnosis and differentiation of STSCH from its mimics [205]. Specific imaging techniques can confirm the presence of soft tissue extending from the apex of the cord displacement through the dural defect into the epidural space, thereby solidifying the diagnosis of STSCH [206]. The degree of cord deformity or kinking observed on imaging can vary widely, which is important for interpretation.

As previously detailed (Table 1) when imaging findings suggest focal cord displacement alongside a widened CSF space, neuroradiologists must consider STSCH and various CSF-isointense space-occupying intraspinal lesions in their differential diagnoses. Increased awareness and advancements in imaging technologies have led to more frequent diagnosis of STSCH in recent years. A multimodal imaging approach, combined with a thorough understanding of clinical symptoms, is essential for accurate diagnosis and timely treatment [58,62,207,208]. A retrospective case series proposed a classification system for imaging findings associated with STSCH [64]. Some studies suggest that dural lysis resulting from a bone defect or disc herniation might play a role [59,63,70,85]. The relationship between Type C classification and bone defects appears to be strongly linked to symptom severity and surgical outcomes [64]. Overall, the combination of improved imaging techniques and heightened clinical awareness is critical for the accurate identification and management of this rare condition.

The MRI and CT myelography images were categorized based on the severity of herniation and displacement (sagittal plane), the herniation level as either vertebral or disc level and presence/absence of bony spurs surrounding the spinal cord herniation (axial plane), as in Table 2.

### 4.5. Spinal Arachnoid Web

Imaging is considered the gold standard for diagnosing SAW, as it typically reveals a scalpel-shaped indentation on the posterior aspect of the spinal cord in the sagittal plane. This sign is a highly specific, though not pathognomonic, indirect indicator of SAW, usually accompanied by a preserved anterior subarachnoid space [71,209,210]. The scalpel sign may present with an abrupt upper point or, less commonly, a more pronounced lower point [106]. This sign can be visualized via MRI or CT myelography, with the latter reserved for cases where MRI fails to provide clarity due to its invasive nature [106,211,212,213]. However, the scalpel sign is not universally present; some arachnoid webs exhibit a “C”-shaped dorsal indentation or, in certain cases, no indentation on MRI [112,134]. CT myelography, while more invasive, may offer enhanced sensitivity in determining the precise location of SAWs, and an air bubble inadvertently introduced into the subarachnoid space during the procedure can become trapped at the level of the SAW, serving as a useful localization clue [213].

Direct visualization of the SAW is possible on MRI in a minority of cases, particularly with 3D T2 sequences or CISS sequences [108,120,149,212]. In these instances, the SAW appears as a thin transverse band located intradurally and extramedullarily, attached to the dorsal aspect of the spinal cord, displaying a hypointense signal on T2 sequences [112,134]. Syringomyelia is frequently associated with SAW, typically extending above the level of the arachnoid web. The location of the syrinx develops above the arachnoid web if the caudorostral flow is impeded, below the web if the rostrocaudal flow is obstructed, and potentially on both sides if both flows are hindered, as indicated by quantitative CSF flow studies performed by Chang et al. [112].

The differential diagnoses for SAW include spinal arachnoid cysts and spinal cord herniation. MRI plays a crucial role in differentiating these conditions. Arachnoid cysts typically display well-defined borders, a gradual filling pattern on MRI cerebrospinal flow imaging or CT myelography, and a smooth spinal cord indentation that does not exhibit the scalpel shape characteristic of arachnoid webs. Artifacts in CSF flow can also help distinguish between arachnoid webs and arachnoid cysts, as they are more pronounced in SAW, indicating obstructions in dynamic flow at the obstruction site, while diminished in arachnoid cysts.

Additionally, MRI CINE sequences and flow sequences can demonstrate blocked CSF or disturbed flow with increased velocities. In contrast, spinal cord herniation is typically marked by an anterior shift of the spinal cord, an interrupted anterior subarachnoid space, and a distorted posterior aspect of the cord resembling the letter “C.” Preservation of the anterior subarachnoid space is often more evident on CT myelography. Investigating these patients may involve extensive preoperative and intraoperative studies to assess the arachnoid webs. MRI is the gold standard investigation, although it has suboptimal sensitivity due to the relatively thin size of the webs compared to adjacent tissue. Yamaguchi noted that it is challenging for MRI to visualize focal arachnoid lesions and can only suggest webs due to spinal cord deformity and obstructed CSF flow [114].

However, high-resolution sagittal T2-weighted imaging can identify several features:

- An extramedullary transverse band of arachnoid tissue extending to the dorsal surface of the spinal cord.
- Dorsal indentation of the spinal cord.

Together, these features comprise the “Scalpel Sign,” which is considered pathognomonic of SAW, in particular in the thoracic spine (thoracic SAW). The term is derived from the mass effect on the dorsal spinal cord resulting from accumulated CSF, resembling a surgical scalpel with its blade pointing posteriorly [106,113]. The scalpel sign is a radiological finding observed on sagittal MRI and CT myelography images, corresponding to the indentation in the dorsal aspect of the spinal cord [106]. Its presence suggests local arachnoid adhesion along the dorsal aspect of the spine and widening of the dorsal subarachnoid space [106]. It is regarded as a pathognomonic imaging discovery linked to thoracic SAWs [106,107,116]. However, other spinal entities, such as spinal arachnoid cysts and ventral spinal intradural cyst herniation, can mimic thoracic SAWs on MRI, potentially leading to misdiagnosis [107,109,122,159]. Imaging plays a significant role in establishing a precise presumptive diagnosis, guiding optimal treatment and the need for a surgical approach.

**Figure 7:**
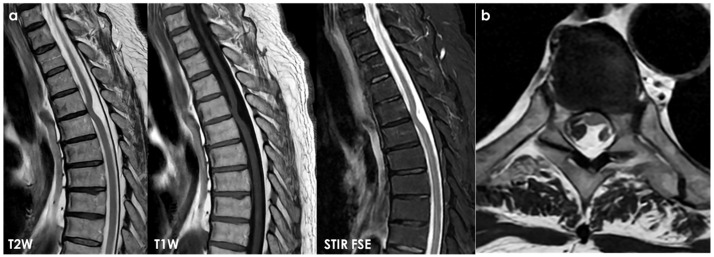
Spinal dorsal MRI in sagittal (**a**) and axial T2W (**b**) sections, showing a classical “scalpel sign”. The diagnosis of SAW was surgically confirmed.

An example of SAW is illustrated in Figure 7.

CT myelography may also miss SAW, as it relies on the principle of obstruction to CSF flow, and thoracic SAWs typically do not cause complete obstruction. Due to the elusive nature of thoracic SAWSAWs in conventional imaging studies, alternative sequences have been employed to enhance sensitivity in diagnosing these conditions.

Conventional MRI sequences may not adequately evaluate the pulsatile movement of structures within the subarachnoid space due to motion artifacts [213,214,215]. For instance, a web was not confidently identified in any of the 14 cases reported by Reardon et al. [107] and was identified in only 2 of 7 cases by Hakky et al. [215]. Improved visualization of these webs may be achieved through high-resolution myelographic T2-weighted MRI sequences such as CISS or SPACE [66,68,128,216]. Additionally, CINE MRI—specifically cardiac-gated phase-contrast cine-mode MRI—has demonstrated improved capability in identifying and correctly localizing the scalpel sign associated with SAW. This technique allows for the observation of a one-way valve-like flow of CSF due to the presence of the web, providing valuable insights into the dynamics of CSF flow around the obstruction [112]. This technique also allows for the evaluation of improved CSF flow following surgical intervention. Overall, these advanced imaging modalities significantly contribute to the accurate diagnosis and localization of arachnoid webs, addressing the limitations of traditional imaging techniques.

**Figure 8:**
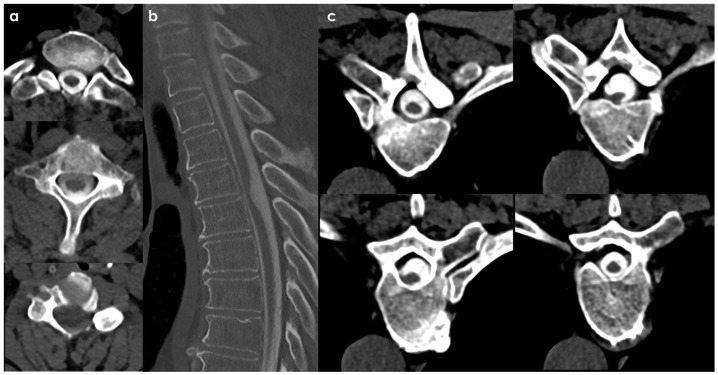
CT Myelography of the same patient as Figure 6, showing axial supine (**a**), sagittal (**b**) and axial prone (**c**) images, showing the eccentric attraction of the spinal cord at the level of the intervertebral discus.

**Figure 9:**
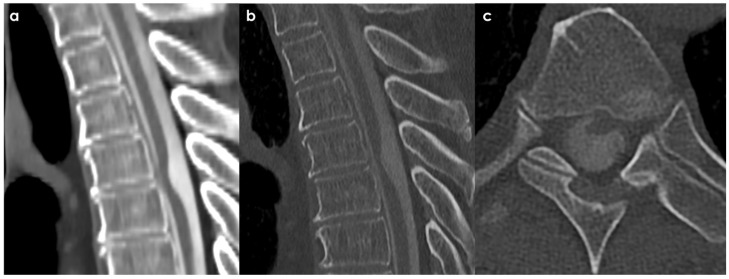
Detail of the sagittal (**a**,**b**) and axial CT Myelography (**c**) with the magnified finding of spinal cord herniation. (**b**,**c**) are in a soft tissue window.

The CT myelography findings are illustrated in Figure 8 and Figure 9.

**Figure 10:**
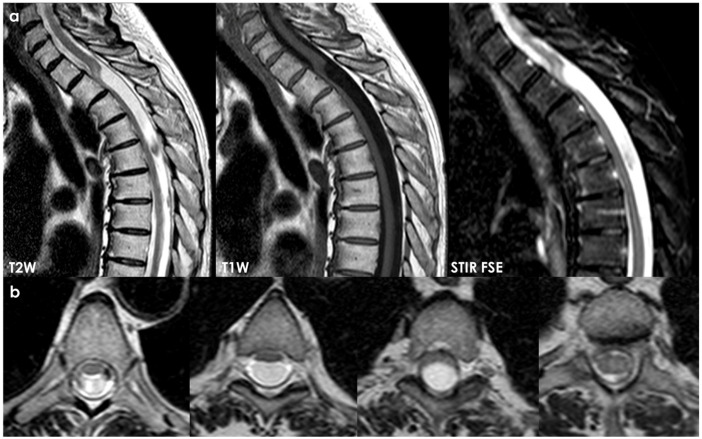
Post-traumatic spinal arachnoid web with scalpel sign and intraspinal lesion. (**a**) shows sagittal MRI and (**b**) the axial sections around the attraction level.

Another example is proposed in Figure 10.

The main neuroradiological differential diagnoses of thoracic SAW are summarized in Table 3.

**Figure 11:**
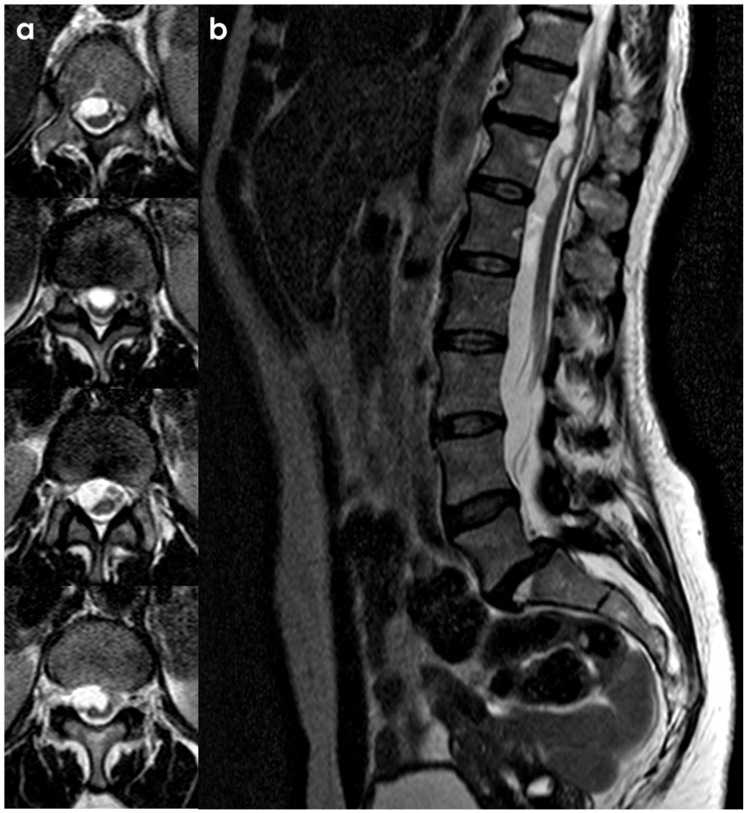
Spinal multiloculated arachnoid cysts with deformation and dislocation of the spinal cord in axial (**a**) and sagittal (**b**) T2W MRI.

As pointed out in the previous section, spinal cord herniations are considered rare events, typically occurring at dorsal locations following trauma or surgery, with the lumbar spine being the most frequent site of occurrence. In contrast, spontaneous ventral herniation of the dorsal spinal cord through a previously uninjured dura is an exceptional phenomenon with an unknown pathogenesis, predominantly manifesting in the thoracic spine [67,203]. SACs and STSCH can coexist and are often attributed to traumatic etiologies. Sporadic displacement and herniation of the spinal cord associated with SAC may result from increased dorsal pressure [67,203]. However, it is important to note that many STSCH believed to be associated with SAC may actually represent STSCH with dorsal enlargement of the CSF space. In fact, there is a common misconception among authors regarding the association between dorsal intradural arachnoid cysts and STSCH. In reality, these cases often involve dorsally enlarged CSF spaces with loose arachnoid membranes resulting from the traction of the spinal cord into a dural defect [67,203,215,216,217,218]. This distinction is crucial for accurate diagnosis and management, as mistaking enlarged CSF spaces for arachnoid cysts can lead to inappropriate treatment approaches. An example of a multilocalulated SAC is in Figure 11.

Although CT myelography and MRI are generally reliable for diagnosing SAWs and related lesions, establishing the precise location of the lesion preoperatively can be challenging due to the fine anatomical structures that may be elusive on standard MRI sequences [112]. MRI typically shows a focally widened dorsal subarachnoid space with ventral displacement of the spinal cord. T2-weighted signal attenuation in the dorsal CSF space may also be observed in arachnoid webs and cysts, linked to obstructive components and disrupted CSF flow. Consequently, the presence of arachnoidal bands or membranes may be associated with changes in cord signal intensity on MRI, potentially leading to the development of syringomyelia in a significant proportion of cases involving thoracic SAWs and SACs [67,116,203,219]. The location of syringomyelia relative to the arachnoid web is believed to occur where intramedullary pulse pressure is lower compared to the opposite side of the web. Klekamp [220] noted that syringomyelia was found rostral to the web in 47% of cases, caudal in 24%, and on both sides in 29% of cases. Differentiating STSCH from SACs or SAWs involves identifying evidence of cord tissue protruding through a ventral dural defect toward the anterior epidural space, with focal ventral deformity and asymmetry of the cord [116,163,203]. Analyzing the presence or absence of CSF signal ventral to the cord on the sagittal plane may also be helpful [216,219,220,221]. Schultz et al. [219] compared radiological features of STSCH and SAW, finding that the presence of a spinal cord segment extending ventrally outside the dura mater was indicative of ventral spinal cord herniation. However, close apposition between the ventral spinal cord and the anterior dura can complicate the differentiation between these entities [221,222].

SACs can be identified by their well-defined margins, smooth contours, and wide scalloping of the cord surface, as they typically fill more slowly than the remainder of the subarachnoid space on myelography [107,108]. Notably, SACs are less frequently associated with syringomyelia compared to thoracic SAWs. Less specific imaging findings, such as vertebral scalloping or widening of the vertebral pedicle, may also be seen in larger, chronic SACs [99]. On axial views, the retromedullary T2 signal in SACs is usually isointense or slightly hyperintense compared to the CSF signal, with variability depending on CSF pulsatility or cyst protein content [66]. In SACs, the spinal cord is often displaced anteriorly and laterally in an asymmetric manner, while thoracic SAW typically shows symmetric anterior displacement, accompanied by hypointense areas in the retromedullary CSF signal, indicating turbulent flow in a widened dorsal subarachnoid space [66].

The differentiation between SAWs and STSCH is particularly crucial, as both are uncommon abnormalities affecting the thoracic spinal cord that can lead to syringomyelia and significant neurological morbidity if left untreated [63,107]. Unfortunately, there are no specific clinical features that distinguish SAWs from STSCH, as both can present with a similar range of symptoms, from subtle sensory and motor deficits to paraparesis and manifestations of Brown–Séquard syndrome. SAWs are characterized by transverse bands of thickened arachnoid membrane that compress the dorsal aspect of the cord [63,107]. In contrast, spinal cord herniation involves focal ventral or, less commonly, lateral protrusions of a segment of the spinal cord through a dural defect [221]. Differentiating these two entities based on clinical presentation and radiological findings is challenging but essential for surgical planning. While surgical access for both lesions is similar, definitive treatment of spinal cord herniation necessitates division of the dentate ligaments to allow for inspection of the ventral cord and dura, whereas thoracic SAW can be treated with lysis of the web without requiring such exposure or repair [219].

Some authors suggest that thoracic SAW and STSCH can be reliably distinguished on imaging by examining the nature of the dorsal indentation and the integrity of the ventral subarachnoid space at the level of the cord deformity. The septum posticum of Schwalbe, which traverses the posterior spinal subarachnoid space, is especially well developed in the thoracic spinal canal and serves to stabilize the cord. It may sometimes appear as a filling defect on supine myelograms [63,223]. This septum is believed to degenerate progressively in the presence of SACs, with webs and adhesions representing remnants that can compress the cord and impede CSF flow to varying degrees [214,224].

## 5. The Need for a Unifying Concept and Strategy

The concept of duropathy as a unifying label for different diseases with similar pathogenesis, anatomical and imaging findings has been proposed and in recent years it has been quietly widened. The hallmark of the cascade of events in these diseases is the presence of dural damage in the ventral spinal cord, i.e., a dural tear. It has been proposed that idiopathic conditions failed to report a previous trauma or trigger, but the natural history is almost the same.

**Figure 12:**
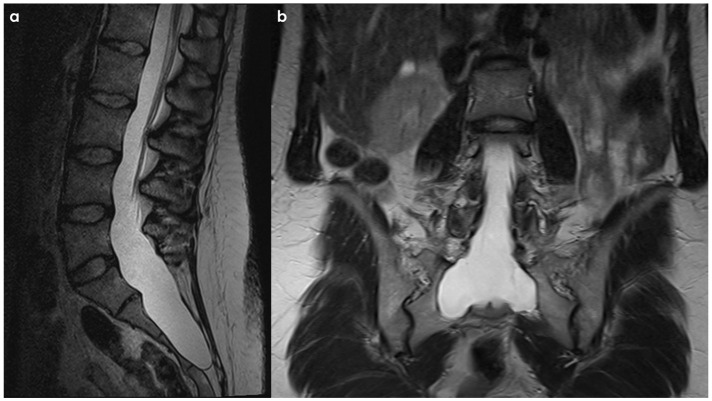
Dural ectasia with megasac in lumbosacral MRI (T2W) in sagittal (**a**) and coronal reconstructed plane (**b**).

Interestingly, a dural “weakness” has been reported in some hereditary connective tissue diseases, such as hypermobile Ehlers-Danlos syndrome (hEDS) [225,226] and Marfan Syndrome (MFS). EDS exhibits a constellation of symptoms across multiple body systems, as well as chronic and acute pain, making daily life challenging for this patient population. Craniospinal neurological manifestations affecting those with hEDS can include scoliosis, CSF leaks, spinal instability, tethered cord syndrome, and Chiari malformation, among others [225]. Spinal CSF leaks are a cause of intracranial hypotension syndrome in EDS patients (cardiac and hypermobile phenotypes) due to the fragility of the dura [226]. MFS is characterized by dural ectasia on imaging [227]. In fact, dural ectasia is one of the major criteria for MFS in the Ghent nosology and has been defined as “enlargement of the neural canal anywhere along the spinal column, but nearly always in the lower lumbar and sacral regions; thinning of the cortex of the pedicles and laminae of the vertebrae; widening of the neural foramina; or an anterior meningocele” [228]. Dural ectasia is also present in other inherited connective tissue diseases. In a long-term follow-up study [229], 52 out of 58 patients with hereditary connective tissue disorders and 11 controls had dural ectasia at follow-up. In particular, 45 MFS patients had dural ectasia at follow-up compared to 41 at baseline; 5 Loeys–Dietz patients showed dural ectasia at follow-up vs. 4 at baseline. Additionally, 24 MFS and 2 Loeys–Dietz patients had anterior sacral meningocele at follow-up, compared with 21 and 1, respectively, at baseline. Notably, three MFS patients developed herniation of a nerve root sleeve during follow-up. An example of sural ectasia in an EDS patient is illustrated in Figure 12.

Currently, no studies have determined the prevalence of monogenic connective tissue diseases using genetic testing, nor have we applied the EDS or MDS diagnostic criteria to patients with hard-tissue diseases. This area therefore remains to be clarified. Likewise, any vascular comorbidities in these patients (e.g., spontaneous arterial dissections or aneurysms) are unknown, as this aspect has never been systematically studied.

In general, several questions remain unanswered. The reason why the same supposed lesion (spinal dural tear) may raise so different clinical manifestations is a matter of debate but no solutions have been proposed. The mechanism by which SS develops in patients with a dural defect and VLISFC is still unclear. Future studies are needed to accurately define the location and pathology of the VLISFC. Furthermore, we lack an explanation for why some patients with a dural defect develop SS while others present with SIH, STSCH, or multisegmental atrophy. In the case of a slowly progressive condition like SS, long-term follow-up is essential to verify that the repair of the dural defect and resolution of the VLISFC are associated with lasting clinical stability.

Another relevant issue is the pathophysiology of intracranial hypotension, in particular SIH, because some long standing cases with a complex and not always readable clinical history may be the other end of the spectrum of intracranial pressure (or, better, volume) diseases, i.e., the shift from idiopathic intracranial hypertension (IIH) to intracranial hypothension through the development of dural leak induced by the long-term increase in intracranial pressure. In fact, IIH may be associated with cranial (not spinal) CSF leaks [229], but the surgical repair of a spinal dural tear causing SIH may provoke rebound intracranial hypertension [230,231]. In addition, a recent report describes the occurrence of SIH and signs due to a cranial CSF leak [232], supporting the hypothesis of a more complex pathophysiology wit overlapping clinical and neuroradiological phenotypes not only at the onset but also during the natural history of duropathies.

Treatment is another relevant issue but final answers are lacking. If a dural tear is identified, surgical intervention is warranted [233,234] and or type III leaks endovascular treatment too should be considered [235]. Due to the rarity of duropathies, no evidence is available for treatment options, with only isolated case reports and case series being present. Therefore, it cannot be clearly stated who should be treated among patients with various subtypes of duropathies, when treatment should be proposed in the natural history of these diseases, and how individual patients should be managed. The literature suggests proposing surgery even for relatively asymptomatic patients with SS and an active leak, as the natural progression of the disorder generally involves slow advancement with potential for irreversible neuronal injury and significant neurological compromise. However, the decision-making process should consider the slowly progressive nature of the disorder, the underlying etiology, the presence of active bleeding, and other medical comorbidities, particularly in elderly patients. Dural defects can be repaired using sutures, patches, collagen sponges, fibrin glue, or muscle/fat grafting. Patients with SS who have dural defects accompanied by intraspinal fluid collections, pseudomeningoceles, or root avulsions often do not benefit from epidural blood patches [236]. This may be attributed to the chronic nature of the process, rapid leaks, large defects, or the presence of associated osteophytes. The meninges adjacent to the defect in SS may exhibit fibrosis, thickening, and discoloration. Innovative approaches, such as spinal endoscopy with CT myelography or intraoperative ultrasonography, have also been utilized to detect and repair small dural defects in SS [237,238,239]. In addition, the assessment of outcomes requires long-term follow-up due to the slowly progressive nature of the primary duropathies.

Given the variability of duropathies the potential overlap in their natural history and manifestations, it is necessary to establish a standardized diagnostic and therapeutic approach. This would allow for the collection of high-quality prospective data in international multicenter contexts, potentially identifying centers and professionals from different backgrounds (vascular neurologists, neuroradiologists, neurosurgeons, etc.) who can serve as references for the referral and management of patients.

## 6. Conclusions

The concept of duropathy serves as a unifying label for various diseases sharing similar pathogenesis, anatomical features, and imaging findings. Central to these conditions is the presence of dural damage in the ventral spinal cord, specifically a dural tear. It has been suggested that idiopathic cases may fail to report prior trauma or triggering events, yet the natural history of these conditions appears remarkably similar. Interestingly, a degree of dural “weakness” has been observed in certain hereditary connective tissue disorders, such as hypermobile EDS and MS. These conditions exhibit a wide array of symptoms across multiple systems, including chronic pain, and can lead to significant craniospinal neurological manifestations, such as scoliosis, CSF leaks, spinal instability, tethered cord syndrome, and Chiari malformation. Spinal CSF leaks are a primary cause of intracranial hypotension in patients with connective tissue disorders, attributed to dural fragility. Despite the rarity of duropathies, their variability and potential overlap in clinical presentations necessitate a standardized diagnostic and therapeutic approach. By establishing a framework for high-quality prospective data collection across international multicenter contexts, it may be possible to identify dedicated centers and professionals from diverse backgrounds—such as vascular neurologists, neuroradiologists, and neurosurgeons—who can facilitate patient referral and management. Future research should focus on clarifying the underlying mechanisms of chronic red blood cell leaks associated with dural tears, the role of biomarkers such as CSF ferritin, and the potential for minimally invasive techniques to repair dural defects. Additionally, long-term follow-up is essential to evaluate the effectiveness of interventions and monitor for long-term sequelae in patients with duropathies.

## Figures and Tables

**Table 1:** Imaging Characteristics of STSCH and CSF-Isointense Intradural Extramedullary Lesions [203].

Disease	T1W	T1W-CE	T2W	CTM	DWI	Clinical Presentation	Comments
STSCH	Anterior displacement of the spinal cord, possible kinking	No enhancement	Anterior displacement of the spinal cord, possible kinking	No filling defect	No restricted diffusion	BSS, paraparesis, isolated sensory or motor weakness	Delayed diagnosis common; may see soft tissue extending through the dura on high-resolution MR images. Flow artifact posterior to the cord suggests absence of a space-occupying mass.
Epidermoid cyst	Iso- to hyperintense	Mild to no peripheral enhancement	Iso- to hyperintense	Filling defect	Restricted diffusion	Nonspecific; signs and symptoms related to tumor size and location	Appearance at T1WI and T2WI depends on cystic protein concentration; hyperintense to CSF on FLAIR MR images.
Arachnoid cyst	Isointense	No enhancement	Iso- to slightly hyperintense	Usually no filling defect	No restricted diffusion	Nonspecific; pain is the most common presenting symptom	Pain may worsen with Valsalva maneuver; may see scalloping of the posterior vertebral body or widening of the pedicles; iso- to hyperintense to CSF on FLAIR MR images.
SEA	Hypo- to slightly hyperintense relative to spinal cord	Diffuse homogeneous or heterogeneous enhancement	Hyperintense relative to spinal cord	CTM not recommended; may seed infection into subarachnoid space	Restricted diffusion	Localized back pain, fever, neurologic deficit	Use of T1WI and T1WI-CE is critical to detection.
Cystic Schwannoma	Hypo- to isointense relative to spinal cord	Peripheral nodular enhancement	Mildly to markedly hyperintense relative to spinal cord	Filling defect	No restricted diffusion	May see T2 signal shine-through artifact; often asymptomatic	Pain or localized findings if growth is large.

**Table 2:** Imaging characterization of SISCH, adapted from [64].

Plane	Type	Features
Sagittal	Kink type (Type K)	It displays a noticeable spinal kink towards the ventral side
	Discontinuous type (Type D)	The spinal cord is completely absent at the herniated site
	Protrusion type (Type P)	It is characterized by the disappearance of the subarachnoid space in the anterior spinal cord with minimal kink in the posterior spinal cord
Sagittal	Vertebral	Herniation at the vertebral level
	Disc	Herniation at the disc level
Sagittal	Bone spurs around the herniation	Present/absent
Axial (for hiatus location)	Central (Type C)	Central hiatus
	Lateral (Type L)	Lateral Hiatus
Axial (for the laterality of herniated spinal cord)	Same (Type S)	Corresponding to the hiatus location
	Opposite (Type O)	On the opposite side of the hiatus location
Axial	Bone defect at the hiatus	Present/absent

**Table 3:** Main neuroimaging features for the differential diagnosis of thoracic SAW, STSCH, and SAC.

Condition	Main Neuroimaging Features
Thoracic SAW	- Focal dorsal indentation of the spinal cord - Increased T2-weighted signal in the spinal cord - Widened dorsal subarachnoid space - Attenuation of CSF signal in the dorsal space
STSCH	- Anterior displacement of the spinal cord - Evidence of cord tissue protrusion through a ventral dural defect - Dorsal subarachnoid space may be narrowed - Increased T2-weighted signal in the spinal cord
SAC	- Anterior displacement of the spinal cord - Distinct margins of the cyst with smooth contours - Increased T2-weighted signal in the cyst - Presence of a well-defined cystic structure adjacent to the spinal cord

## Data Availability

No new data were produced in this paper.
